# Trends and characteristics of multiple births in Baoan Shenzhen: A retrospective study over a decade

**DOI:** 10.3389/fpubh.2022.1025867

**Published:** 2022-12-13

**Authors:** Wenyi Tang, Lingyun Zou

**Affiliations:** ^1^Department of Clinical Data Research, Chongqing University Central Hospital, Chongqing Emergency Medical Center, Chongqing, China; ^2^Shenzhen Baoan Women's and Children's Hospital, Jinan University, Shenzhen, China

**Keywords:** multiple births, secular trend, risk factor, very low birth weight, Apgar score, height/head circumference ratio

## Abstract

**Background:**

Shenzhen has the largest and youngest foreign population among all cities in China. The reproductive health of pregnant women from different backgrounds is a social issue that deserves attention. In the past decade, China has liberalized its population policies to stimulate population growth, and the proportion of multiple births has continued to increase.

**Method:**

This retrospective cohort included 526,654 newborns born in Baoan, Shenzhen, from January 1, 2009, to December 31, 2019, including 515,016 singletons and 11,638 twins or triplets. Univariate regression models were used to analyze the effects of maternal sociodemographic characteristics, physiological characteristics, medical history, antenatal care and other factors associated with single vs. multiple births and to elucidate the changing trends of different factors affecting multiple births in the past 11 years. Additionally, fetal development in multiple births was analyzed by generalized linear mixed models.

**Results:**

The rates of pregnancy complications, preterm birth, and advanced-age pregnancy were significantly higher in the multiple birth mothers than in single birth mothers, and more multiple pregnancies were achieved through assisted reproductive technologies. The rates of adverse outcomes such as stillbirth, malformation, hypoxia, and ultralow body weight in multiple fetuses were significantly higher than that in singleton fetuses. The trend analysis from 2009 to 2019 showed that the socioeconomic status and health level of mothers with multiple births improved over time, and the risk during pregnancy generally decreased. Simultaneously, the development indicators of multiple fetuses have improved year by year, and the proportion of adverse outcomes has also decreased significantly. A low pre-natal care utilization rate was shown to be detrimental to the development of multiple fetuses. Independent risk factors for hypoxia and very low birth weight were also identified. The differences in secular trends between two birth groups were further revealed by time series models.

**Conclusion:**

This study presented a comprehensive survey of multiple pregnancies in the area with the largest population inflow in China. This study identified the factors that affect the health of multiple birth mothers and their fetuses, particularly suggesting that preterm birth rates and the use of assisted reproduction remain high. The findings provide a basis for the formulation of individualized pre-natal care, assisted reproductive guidance and healthcare policies for multiple births.

## 1. Introduction

A multiple birth is the culmination of one multiple pregnancy, wherein the mother gives birth to two or more offspring that were gestated simultaneously. The rate of multiple births in developed countries has continued to rise over the past four decades, countries such as England, Wales, France, and the United States have all seen a 50–60% increase in twin birth rates (1–3), the global twin birth rate has risen by a third, from 9 per 1,000 to 12 per 1,000 today, and triplet, quadruplet, and high-order multiple births have increased at an even faster rate (4, 5). In China, multiple pregnancies have not received much attention during the long era of restrictive family planning policies (6, 7). Since China proposed liberalizing the two-child policy in 2014, the rate of multiple births has shown an upwards trend. Although major medical advances have improved the outcomes of multiple births, multiple births are still associated with significant medical risks and complications for the mother and children. The rates, causes and risks of multiple pregnancy require more attention.

In recent years, issues related to multiple pregnancy, such as fertility, preterm birth, congenital pancreatitis, low birth weight, risk of cerebral palsy, and assisted reproductive technology, have received increasing attention from international community organizations and research institutions (8–11). In 2020, the WHO released a series of new recommendations to improve the quality of pre-natal care to reduce the risk of stillbirth and pregnancy complications (12). In 2021, to promote the long-term balanced development of the population, China revised the “Population and Family Planning Law,” formally implemented the three-child policy, and committed to continuously improving the health of women and children (13). Although increasing attention has been given to pregnant women at home and abroad, there are very few large-scale retrospective studies specifically aimed at evaluating the risks of multiple fetus intrauterine development and asphyxia during delivery, and the updates of various policies rarely give separate consideration to pregnant women with multiple births. With the implementation of the three-child policy, China may face more challenges of multiple births.

Shenzhen is one of the fastest growing cities in China, with a large floating and young population (14), and a considerable number of babies are born every year. In Baoan District, Shenzhen, there are more than 6 million permanent residents, and more than 50,000 babies are born every year. The government needs to constantly optimize policies to meet the huge needs of maternal and infant healthcare. Therefore, we selected the maternal data of this region in the past 11 years, focused on analyzing the factors influencing multiple pregnancy and fetal development, and revealed the secular trends of multiple pregnancy. We expect this study to provide a reference for optimizing healthcare policies and approaches to multiple pregnancies.

## 2. Materials and methods

### 2.1. Study population

This cohort study was based on data including all births in Baoan from 1 January 2009 to 31 December 2019, extracted from the Shenzhen Birth Registry Database, which has served as a system for birth registration and maternal and infant health management since 2000. The data are based on the observations of the fetuses, and the number of mothers includes duplications due to multiple birth records (15). We collected 526,654 newborns born in Baoan, Shenzhen, from January 1, 2009, to December 31, 2019, including 515,016 singletons and 11,638 twins or triplets. This standardized and structured study collected a variety of information extracted from medical records about demographic factors, reproductive and medical history, environmental and lifestyle factors, birth outcomes and maternal complications. We compiled maternal sociodemographic and physiological characteristics and infant clinical records for the analysis of fetal development and other relevant aspects.

### 2.2. Definition

The Apgar score is a quick method to assess a newborn's post-natal and resuscitation response and has been endorsed by the American College of Obstetricians and Gynecologists and the American Academy of Pediatrics. Neonatal asphyxia was defined as an Apgar score ≤7 within 1 or 5 min. Very low birth weight (VLBW) [8] infants were defined as infants born with a body weight <1,500 g. Non-VLBW infants were those with a birth weight ≥1,500 g. The height/head circumference is defined as the ratio of the height to the head circumference. According to the reference value of the height/head circumference ratio of Chinese newborns, 80% of the newborns have a height/head circumference between 1.35 (10%) and 1.55 (90%) (16). This ratio balances the impact of pregnancy on individual aspects of fetal head and body development. In this study, fetuses with a height/head circumference outside the range of [1.35, 1.55] were removed to allow for a focused analysis. Pre-natal care visits were converted into pre-natal care utilization by calculating the ratio of actual visits to the number of visits recommended by the WHO (17). It was then classified into four categories: low (<50%), substandard (50–80%), close to recommended (80–110%), and appropriate (≥110%). Post-partum hemorrhage was defined as bleeding of more than 500 ml during natural delivery or 1,000 ml during cesarean section. Assisted reproductive technology (ART) (18–21) refers to producing a pregnancy for an infertile couple by medical assistance, including artificial insemination (AI), *in vitro* fertilization and embryo transfer (IVF-ET) and their various derivatives.

### 2.3. Statistical analysis

Numbers and frequencies (%) are reported for categorical variables, and medians (Q1, Q3) are reported for continuous variables with a non-normal distribution or uneven variance. Pearson's chi-square tests or Fisher's exact test were used to compare the selected characteristics between single births and multiple births. Unconditional logistic regression models were used to estimate the odds ratio (OR) and 95% confidence interval (95% CI) for categorical outcomes. Significant variables found in univariate analysis were incorporated into the multiple logistic regression model to obtain independent factors influencing multiple births.

Univariate analysis was used to analyze the influence of maternal pregnancy socioeconomic indicators, physiological status, pregnancy and childbirth history, living habits, etc., on singleton and multiple births (one mother was observed once, and any multiple records were deleted for repeated multiple births) and used to analyze the distribution difference of fetal development between multiple births and single births (one observation of the fetus was carried out). Multivariate analysis with a generalized linear mixed model (GLMM) was used to study the effects of maternal pregnancy socioeconomic and physiological factors on fetal ultralight birth weight, length-to-head circumference ratio, and asphyxia at 1 min and 5 min.

Sliding-window time series models were adopted to analyze secular characteristics of maternal and fetal metrics. The difference significance between the time trend slopes were tested by using ANOVA test in a generalized linear model.

All analyses were conducted using R 4.2.0. Alpha levels of 0.001 and 0.05 indicate statistical significance for a two-tailed test separately. Missing values of several variables were included in the descriptive analysis but were removed from the logistic regression analysis.

## 3. Results

From 2009 to 2019, 525,606 pregnant women in Baoan, Shenzhen, were included in the birth population registration database of Shenzhen, and 531,523 babies were born, including 519,714 singletons, 5,867 pairs of twins and 25 pairs of triplets. We excluded 73 pregnant women with a gestational age of <24 weeks, 176 pregnant women with a fetal weight <500 g, and 4,060 pregnant women with incomplete fetal development indicators and ultimately retained 520,860 pregnant women and 526,654 newborns. All collected indicators were manually reviewed to ensure accuracy.

### 3.1. Comparison of demographic characteristics between mothers with multiple births and single births

We performed a comparative analysis of maternal sociodemographic characteristics, health status, and delivery indicators, as shown in Table 1 and Supplementary Table S1. Since 2015, the proportion of multiple births has increased significantly every year compared with 2009, increasing from 0.95% in 2009 to 1.52% in 2019 and from 1% before the two-child policy to 1.35% after the policy. More than three-quarters of multiple birth mothers gave birth between the ages of 23 and 35 (4,426/5,844), while the proportion of older mothers reached 18.1% (1,056/5,844), which was significantly higher than that of single birth mothers (10.9%). Multiple births accounted for 1.31% of the women who gave birth for the first time but decreased to 0.91% for the second time. Most mothers with multiple births received 6–12 years of education, while 31.8% had a college education, compared with only 26.7% of single birth mothers. Obviously, multiple births are more likely to be conceived through assisted reproductive technology than single births (OR = 47.91). The pre-natal care utilization rate of multiple births is higher, with 45.1% of multiple births using 80–100% and above, compared to only 37.5% for single births. In addition, 75.0% of mothers with multiple births belonged to the high-risk group, while only 32.3% of mothers with singletons fell into this category. Multiple pregnancies also caused higher rates of complications for their mothers (OR = 3.41), hypertension (OR = 3.23), eclampsia (OR = 5.83), anemia (OR = 1.63), cesarean section (OR = 10.43), massive hemorrhage (OR = 3.87) and other emergencies. Conspicuously, 46.2% of multiple birth mothers had a premature birth event (2,700/5,844), which was 41.1% higher than that of singleton birth mothers (5.07%). There were no statistically significant differences in the distribution of maternal GBS infections and deaths.

**Table 1:** Monofactor analysis for mothers with multiple and single births in Baoan Shenzhen, 2009–2019.

	**Total**	**Single birth**	**Multiple births**	**P**	**OR (95%CI)**
	**(*N* = 520,860, %)**	**(*N* = 515,016, %)**	**(*N* = 5,844, %)**		
**Year of delivery**					
2009	37,809 (7.26)	37,451 (7.27)	358 (6.13)	–	–
2010	41,039 (7.88)	40,681 (7.90)	358 (6.13)	0.271	0.92 (0.79, 1.07)
2011	45,747 (8.78)	45,338 (8.80)	409 (6.99)	0.426	0.94 (0.82, 1.09)
2012	54,243 (10.41)	53,773 (10.44)	470 (8.04)	0.204	0.91 (0.8, 1.05)
2013	46,315 (8.89)	45,842 (8.9)	473 (8.09)	0.278	1.08 (0.94, 1.24)
2014	49,554 (9.51)	49,027 (9.52)	527 (9.02)	0.088	1.12 (0.98, 1.29)
2015	45,300 (8.70)	44,759 (8.69)	541 (9.26)	0.001	1.26 (1.11, 1.45)
2016	50,539 (9.70)	49,905 (9.69)	634 (10.85)	<0.001	1.33 (1.17, 1.52)
2017	52,121 (10.01)	51,462 (9.99)	659 (11.28)	<0.001	1.34 (1.18, 1.53)
2018	48,433 (9.31)	47,773 (9.28)	660 (11.29)	<0.001	1.45 (1.27, 1.65)
2019	49,760 (9.55)	49,005 (9.52)	755 (12.92)	<0.001	1.61 (1.42, 1.83)
**Two-child policy**					
Before	340,680 (65.41)	337,275 (65.49)	3,405 (58.26)	–	–
After	180,180 (34.59)	177,741 (34.51)	2,439 (41.74)	<0.001	1.36 (1.29, 1.43)
Maternal age^*^					
<19	6,012 (1.15)	5,982 (1.16)	30 (0.51)	<0.001	0.45 (0.31, 0.64)
19–23	54,586 (10.48)	54,244 (10.53)	342 (5.85)	<0.001	0.57 (0.51, 0.63)
23–35	402,683 (77.31)	39,8267 (77.33)	4,416 (75.56)	–	–
35–40	48,649 (9.34)	47,809 (9.28)	840 (14.37)	<0.001	1.58 (1.47, 1.71)
≥40	8,732 (1.68)	8,516 (1.65)	216 (3.7)	<0.001	2.29 (1.99, 2.62)
Parity^*^					
1	241,025 (46.27)	237,877 (46.19)	3,148 (53.87)	–	–
2	226,954 (43.57)	224,897 (43.67)	2,057 (35.2)	<0.001	0.69 (0.65, 0.73)
3	44,410 (8.53)	43,880 (8.52)	530 (9.07)	0.053	0.91 (0.83, 1.00)
≥4	6,333 (1.22)	6,249 (1.21)	84 (1.44)	0.888	1.02 (0.81, 1.25)
**Hypertension**					
No	498,414 (95.69)	493,297 (95.78)	5,117 (87.56)	–	–
Yes	22,446 (4.31)	21,719 (4.22)	727 (12.44)	<0.001	3.23 (2.98, 3.49)
**Education degree (years)**					
<6	10,286 (1.97)	10,179 (1.98)	107 (1.83)	0.011	0.77 (0.63, 0.94)
6~12	371,525 (71.33)	367,649 (71.39)	3,876 (66.33)	<0.001	0.78 (0.74, 0.82)
>12	139,049 (26.70)	137,188 (26.63)	1,861 (31.84)	–	–
**Reproductive techniques**					
No	519,823 (99.80)	514,330 (99.87)	5,493 (93.99)	–	–
Yes	1,037 (0.20)	686 (0.13)	351 (6.01)	<0.001	47.91 (41.97, 54.59)
Pre-natal care utilization rate^*^					
≤ 50%	108,532 (20.84)	107,550 (20.88)	982 (16.80)	<0.001	0.65 (0.6, 0.71)
50–80%	216,743 (41.61)	214,517 (41.65)	2,226 (38.09)	<0.001	0.74 (0.69, 0.8)
80–110%	70,629 (13.56)	69,654 (13.52)	975 (16.68)	–	–
>110%	124,888 (23.98)	123,229 (23.93)	1,659 (28.39)	0.337	0.96 (0.89, 1.04)
**Surgical indication**					
No	330,895 (63.53)	329,868 (64.05)	1,027 (17.57)	–	–
Yes	189,965 (36.47)	185,148 (35.95)	4,817 (82.43)	<0.001	8.36 (7.81, 8.95)
**Complication**					
No	442,014 (84.86)	438,355 (85.11)	3,659 (62.61)	–	–
Yes	78,846 (15.14)	76,661 (14.89)	2,185 (37.39)	<0.001	3.41 (3.24, 3.6)
**Eclampsia**					
No	514,840 (98.84)	509,352 (98.90)	5,488 (93.91)	–	–
Yes	6,020 (1.16)	5,664 (1.10)	356 (6.09)	<0.001	5.83 (5.22, 6.5)
**GBS infection**					
No	519,821 (99.8)	513,986 (99.8)	5,835 (99.85)	–	–
Yes	1,039 (0.2)	1,030 (0.2)	9 (0.15)	0.435	0.77 (0.37, 1.39)
**Anemia**					
No	516,039 (99.07)	510,282 (99.08)	5,757 (98.51)	–	–
Yes	4,821 (0.93)	4,734 (0.92)	87 (1.49)	<0.001	1.63 (1.31, 2)
**High risk**					
No	352,621 (67.7)	351,158 (68.18)	1,463 (25.03)	–	–
Yes	168,239 (32.3)	163,858 (31.82)	4,381 (74.97)	<0.001	6.42 (6.05, 6.81)
**Emergency**					
No	519,822 (99.8)	514,027 (99.81)	5,795 (99.16)	–	–
Yes	1,038 (0.2)	989 (0.19)	49 (0.84)	<0.001	4.39 (3.25, 5.79)
**Pregnancy week**					
<28	317 (0.06)	266 (0.05)	51 (0.87)	<0.001	29.5 (21.59, 39.51)
28–37	29,213 (5.61)	26,564 (5.16)	2,649 (45.33)	<0.001	15.34 (14.55, 16.18)
37–42	485,797 (93.27)	482,660 (93.72)	3,137 (53.68)	–	–
≥42	5,533 (1.06)	5,526 (1.07)	7 (0.12)	<0.001	0.19 (0.08, 0.38)
**Delivery mode**					
Cesarean section	172,971 (33.21)	168,098 (32.64)	4,873 (83.38)	<0.001	10.43 (9.74, 11.18)
Vaginal delivery	347,772 (66.77)	346,808 (67.34)	964 (16.50)	–	–
Else	117 (0.02)	110 (0.02)	7 (0.12)	<0.001	22.89 (9.64, 45.74)
Post-partum hemorrhage^*^					
No	515000 (98.87)	509,395 (98.90)	5,605 (95.90)	–	–
Yes	5,855 (1.12)	5,616 (1.09)	239 (4.09)	<0.001	3.87 (3.38, 4.4)
**Maternal outcome**					
No	520,815 (99.99)	514,972 (99.99)	5,843 (99.98)	–	–
Yes	45 (0.01)	44 (0.01)	1 (0.02)	0.492	2 (0.11, 9.17)

* The missing values of these terms are shown in Supplementary Table S1.

We carefully analyzed and compared various developmental conditions of multiple fetuses and singleton fetuses and listed the results in Table 2 and Supplementary Table S2. During the past 11 years, the proportion of multiple births has increased yearly since 2013 compared with 2009 (*p* < 0.05). During this period, the male-to-female ratio was 1.18 for singleton births and 1.11 for multiple births. For multiple births, the mortality rate (OR = 0.140, 7-day mortality OR = 3.66), malformation rate (OR = 2.01) and neonatal asphyxia rate (Apgar score at 1 and 5 min) were higher than those of single births. Additionally, the results of stratified analysis showed that the main intrauterine growth and development indicators of multiple fetuses were lower than those of singleton fetuses, including weight, height, head circumference, chest circumference, the height/head circumference ratio and the weight/head circumference ratio.

**Table 2:** Monofactor analysis for newborns from multiple births and singleton in Baoan Shenzhen, 2009–2019.

	**Total**	**Single birth**	**Multiple births**	**P**	**OR (95%CI)**
	**(*N* = 526,654, %)**	**(*N* = 515,016, %)**	**(*N* = 11,638, %)**		
**Year of delivery**					
2009	38,150 (7.24)	37451 (7.27)	699 (6.01)	–	–
2010	41,395 (7.86)	40,681 (7.90)	714 (6.14)	0.252	0.94 (0.85, 1.04)
2011	46,152 (8.76)	45,338 (8.80)	814 (6.99)	0.456	0.96 (0.87, 1.07)
2012	54,709 (10.39)	53,773 (10.44)	936 (8.04)	0.167	0.93 (0.84, 1.03)
2013	46,790 (8.88)	45,842 (8.9)	948 (8.15)	0.042	1.11 (1, 1.22)
2014	50,078 (9.51)	49,027 (9.52)	1,051 (9.03)	0.005	1.15 (1.04, 1.27)
2015	45,838 (8.71)	44,759 (8.69)	1,079 (9.27)	<0.001	1.29 (1.17, 1.42)
2016	51,170 (9.72)	49,905 (9.69)	1,265 (10.87)	<0.001	1.36 (1.24, 1.49)
2017	52,776 (10.02)	51,462 (9.99)	1,314 (11.29)	<0.001	1.37 (1.25, 1.5)
2018	49,090 (9.32)	47,773 (9.28)	1,317 (11.32)	<0.001	1.48 (1.35, 1.62)
2019	50,506 (9.59)	49,005 (9.52)	1,501 (12.89)	<0.001	1.64 (1.5, 1.8)
**Two-child policy**					
before	344,054 (65.33)	337,275 (65.49)	6,779 (58.25)	–	–
after	182,600 (34.67)	177,741 (34.51)	4,859 (41.75)	<0.001	1.36 (1.31, 1.41)
**Pregnancy week**					
<28	366 (0.07)	266 (0.05)	100 (0.86)	<0.001	29.01 (22.93, 36.43)
28–37	31,835 (6.05)	26,564 (5.16)	5,271 (45.29)	<0.001	15.31 (14.73, 15.92)
37–42	488,914 (92.83)	482,660 (93.72)	6,254 (53.74)	–	–
≥42	5,539 (1.05)	5,526 (1.07)	13 (0.11)	<0.001	0.18 (0.1, 0.3)
Gender^*^					
Male/female	1.17	1.18	1.11		
Male	284,471 (54.01)	278,353 (54.05)	6,118 (52.57)	–	–
Female	242,173 (45.98)	236,653 (45.95)	5,520 (47.43)	0.002	1.06 (1.02, 1.1)
Fetal position^*^					
LOA	437,871 (83.14)	429,077 (83.32)	8,794 (75.56)	–	–
LSA	12,650 (2.41)	11,711 (2.27)	939 (8.07)	<0.001	3.91 (3.65, 4.19)
ROA	20,299 (3.85)	20,000 (3.88)	299 (2.57)	<0.001	0.73 (0.65, 0.82)
RSA	1,164 (0.22)	985 (0.19)	179 (1.54)	<0.001	8.87 (7.53, 10.38)
Tire	4,008 (0.76)	3,890 (0.76)	118 (1.02)	<0.001	1.48 (1.22, 1.77)
Else	31,295 (5.94)	30,552 (5.93)	743 (6.38)	<0.001	1.19 (1.1, 1.28)
Neonatal outcome^*^					
Born alive	524,388 (99.57)	512,835 (99.58)	11,553 (99.27)	–	–
Early neonatal death	223 (0.04)	206 (0.04)	17 (0.15)	<0.001	3.66 (2.15, 5.82)
Stillbirth	1,703 (0.32)	1,651 (0.32)	52 (0.45)	0.018	1.4 (1.05, 1.82)
**Malformation**					
No	516,504 (98.07)	505,299 (98.11)	11,205 (96.28)	–	–
Yes	10,150 (1.93)	9,717 (1.89)	433 (3.72)	<0.001	2.01 (1.82, 2.21)
**Apgar score at 1 min**					
0–3	2,590 (0.49)	2,483 (0.48)	107 (0.92)	<0.001	1.95 (1.59, 2.35)
4–7	4,123 (0.78)	3,856 (0.75)	267 (2.29)	<0.001	3.13 (2.75, 3.54)
8–10	519,941 (98.73)	508,677 (98.77)	11,264 (96.79)	–	–
**Apgar score at 5 min**					
0–3	2,194 (0.42)	2,115 (0.41)	79 (0.68)	<0.001	1.67 (1.32, 2.07)
4–7	1,025 (0.19)	942 (0.18)	83 (0.71)	<0.001	3.93 (3.12, 4.89)
8–10	523,435 (99.39)	511,959 (99.41)	11,476 (98.61)	–	–
**Weight**					
<1,500	2,965 (0.56)	2,399 (0.47)	566 (4.86)	<0.001	18.86 (17.13, 20.72)
1,500–2,500	23,994 (4.56)	18,788 (3.65)	5,206 (44.73)	<0.001	22.15 (21.28, 23.05)
2,500–4,000	474,433 (90.08)	468,570 (90.98)	5,863 (50.38)	–	–
≥4,000	25,262 (4.8)	25,259 (4.9)	3 (0.03)	<0.001	0.01 (0.00, 0.02)
**Height (cm)**					
<28 Mean (SD)	34.04 (3.09)	34.33 (3.24)	33.29 (2.5)	0.836	1.009 (0.924, 1.105)
Median [Q1, Q3]	34 [32, 36]	35 [32, 36]	33 [31, 35]		
28–37 Mean (SD)	45.99 (3.85)	46.16 (3.89)	45.11 (3.56)	<0.001	1.613 (1.599, 1.627)
Median [Q1, Q3]	47 [44, 49]	47 [45, 49]	46 [43, 48]		
37–42 Mean (SD)	50.06 (1.27)	50.09 (1.25)	48.11 (1.84)	<0.001	1.613 (1.599, 1.627)
Median [Q1, Q3]	50 [50, 51]	50 [50, 51]	48 [47, 49]		
≥42 Mean (SD)	50.49 (1.4)	50.49 (1.4)	49.08 (1.26)	<0.001	1.613 (1.599, 1.627)
Median [Q1, Q3]	50 [50, 51]	50 [50, 51]	49 [48, 50]		
**Head circumference (cm)**					
<28 Mean (SD)	24.72 (2.59)	24.73 (2.61)	24.67 (2.57)	0.116	3.196 (0.76, 13.897)
Median [Q1, Q3]	25 [23, 26]	25 [23, 26]	25 [23, 26]		
28–37 Mean (SD)	31.52 (2.34)	31.56 (2.38)	31.34 (2.13)	<0.001	2028.382 (1479.079, 2779.825)
Median [Q1, Q3]	32 [30, 33]	32 [30, 33]	32 [30, 33]		
37–42 Mean (SD)	33.88 (1)	33.89 (0.99)	32.83 (1.19)	<0.001	2028.382 (1479.079, 2779.825)
Median [Q1, Q3]	34 [33, 34]	34 [33, 34]	33 [32, 34]		
≥42 Mean (SD)	34.12 (1.1)	34.12 (1.1)	33.77 (0.93)	<0.001	2028.382 (1479.079, 2779.825)
Median [Q1, Q3]	34 [34, 35]	34 [34, 35]	34 [33, 34]		
**Height/head circumference**					
<28 Mean (SD)	1.39 (0.13)	1.4 (0.14)	1.36 (0.12)	0.004	1.122 (1.038, 1.216)
Median [Q1, Q3]	1.38 [1.32, 1.46]	1.4 [1.32, 1.48]	1.36 [1.295, 1.43]		
28–37 Mean (SD)	1.46 (0.08)	1.46 (0.08)	1.44 (0.09)	<0.001	1.387 (1.38, 1.394)
Median [Q1, Q3]	1.47 [1.43, 1.5]	1.47 [1.44, 1.5]	1.45 [1.39, 1.48]		
37–42 Mean (SD)	1.48 (0.04)	1.48 (0.04)	1.46 (0.05)	<0.001	1.387 (1.38, 1.394)
Median [Q1, Q3]	1.47 [1.46, 1.5]	1.47 [1.46, 1.5]	1.47 [1.44, 1.48]		
≥42 Mean (SD)	1.48 (0.04)	1.48 (0.04)	1.45 (0.05)	<0.001	1.387 (1.38, 1.394)
Median [Q1, Q3]	1.47 [1.46, 1.5]	1.47 [1.46, 1.5]	1.45 [1.44, 1.5]		
**Weight/head circumference**					
<28 Mean (SD)	39.67 (6.78)	40.44 (6.95)	37.62 (5.85)	0.002	1.002 (1.001, 1.003)
Median [Q1, Q3]	39.2 [35, 43.75]	40.4 [35.42, 45.2]	37.5 [33.265, 41.55]		
28–37 Mean (SD)	76.76 (14.51)	77.77 (14.69)	71.64 (12.39)	<0.001	1.002 (1.002, 1.002)
Median [Q1, Q3]	78.13 [67.93, 86.67]	79.17 [68.97, 87.5]	72.81 [64.14, 80.3]		
37–42 Mean (SD)	99.8 (10.84)	100.01 (10.7)	83.57 (9.41)	<0.001	1.002 (1.002, 1.002)
Median [Q1, Q3]	100 [92.42, 106.25]	100 [93.33, 106.25]	83.33 [77.5, 90]		
≥42 Mean (SD)	105.34 (11.68)	105.38 (11.65)	88.8 (9.85)	<0.001	1.002 (1.002, 1.002)
Median [Q1, Q3]	105.71 [96.97, 112.5]	105.88 [96.97, 112.5]	89.06 [84.12, 96.77]		

* The missing values of these terms are shown in Supplementary Table S2.

### 3.2. Secular trends of multiple births in Baoan, Shenzhen

**Figure 1:**
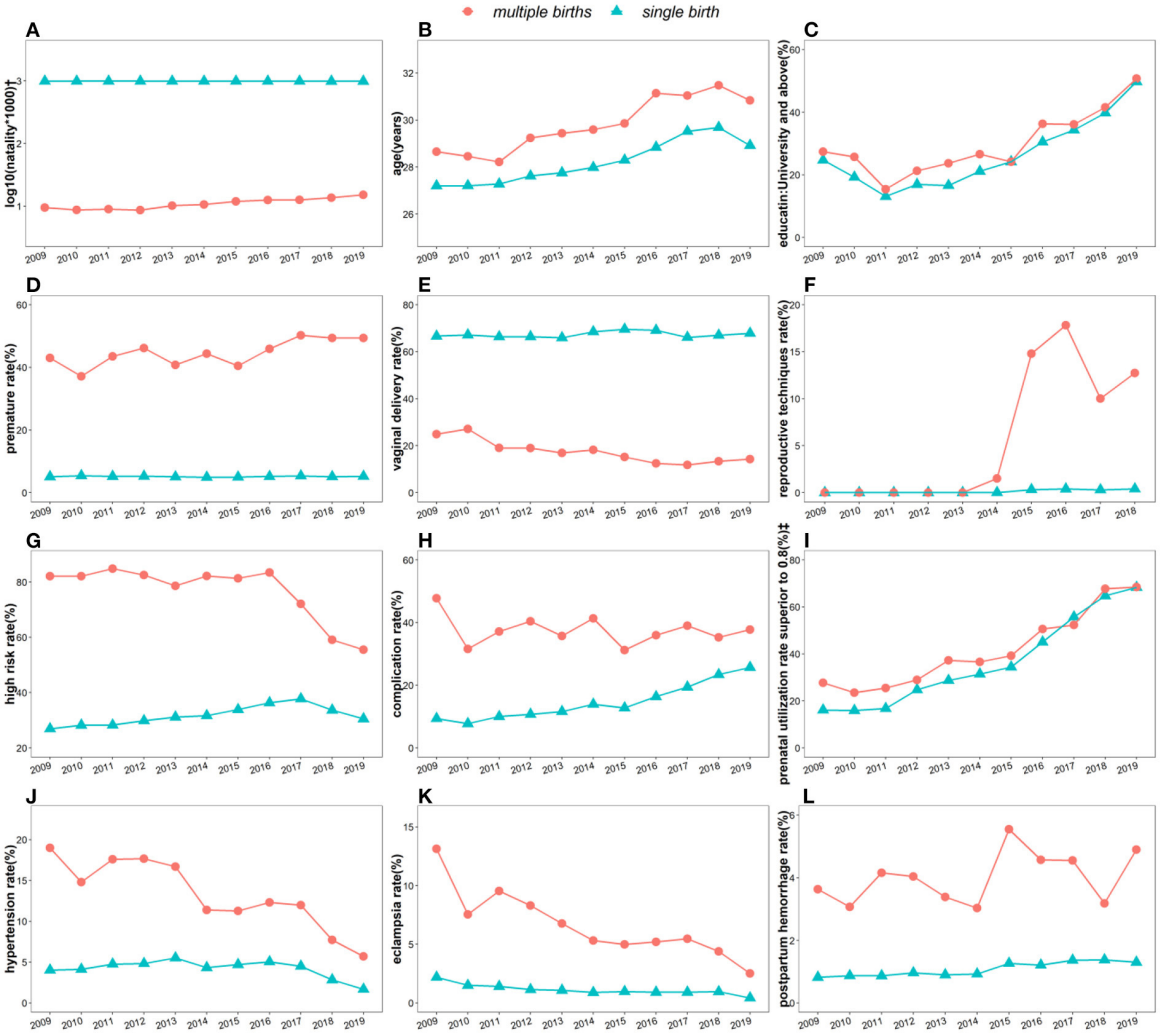
Secular trends of maternal socioeconomic and biomedical characteristics among 520,860 participants sub-categorized by singletons and multiple births in Baoan, Shenzhen, 2009–2019. * **(A)** Natality, **(B)** maternal age, **(C)** education level, **(D)** premature birth rate (<37 week), **(E)** vaginal delivery rate, **(F)** pregnancy rate by assisted reproductive technology, **(G)** high risk factors during pregnancy, **(H)** maternal complications rate, **(I)** pre-natal care utilization rate >80% during pregnancy, **(J)** antenatal hypertension **(K)** eclampsia rate, **(L)** post-partum hemorrhage rate.^†^One puerpera giving birth to twins or triplets only counts as one multiple pregnancy.^‡^Pre-natal care utilization rate is defined as the ratio between the actual number of visits and the recommended number.

We mapped the trends of maternal demographic characteristics, pregnancy-related disorders and complications over the past 11 years. The results of the annual trends are shown in Figure 1. In addition, the secular trends for single and multiple births were analyzed separately using a sliding-window time series model (Supplementary Figure S1). The window width is determined by the decreasing search, and a width of 30 yields the ideal trend curve. Differences in the slopes of the time trend curves between groups were further calculated using a generalized linear model. As shown in Figure 1, the proportion of multiple births has been increasing, from 11.94‰ in 2015 to 15.17‰ in 2019, with an average annual increase of ~0.81‰. The average age of childbearing has gradually increased since 2015 (Figure 1). In particular, as shown in Supplementary Figure S1, the proportion of older parturients has increased yearly and has been advanced by nearly 4 years over the past 11 years, and the average childbearing age of women with multiple births was 2 years older than that of single births. It is particularly noteworthy that 18.07% (1,056/5,844) of multiple birth mothers were ≥35 years old, while only 10.94% (56,325/515,016) were single birth mothers. The proportion with a pre-natal care utilization rate >80% is also increasing yearly, and in 2019, it was nearly four times that in 2009. The rate of premature births among multiple births (gestational age ≤37 weeks) was 10 times higher than that of single births. The proportion of premature infants in multiple births has remained at a high level for over a decade and exceeded 50% for the first time in 2017. The cesarean section rate of multiple births has exceeded 80% since 2016, which is twice that of singletons.

After the full implementation of the two-child policy in 2015, the use of assisted reproductive technology grew significantly. The proportion of mothers with a college education or above has increased, exceeding 55% in 2019 and doubling over 10 years. The incidences of maternal high-risk, hypertension and eclampsia during pregnancy showed a downwards trend, but this trend in multiple births was much higher and declined faster than in single births. Complications and post-partum hemorrhage had a similar trend both in multiple births and in single births, with small fluctuations in multiple births and an upwards trend in single births. In the past 10 years, the incidence of pregnancy complications increased from 9.35% in 2009 to 25.67% in 2019. Among the maternal indicators included in the statistics, except for the education level, all trend curve slopes of multiple births were significantly different with single births (*P* < 0.05). And the *p*-values of these slopes other than post-partum hemorrhage were all <0.001 (Supplementary Figure S1).

**Figure 2:**
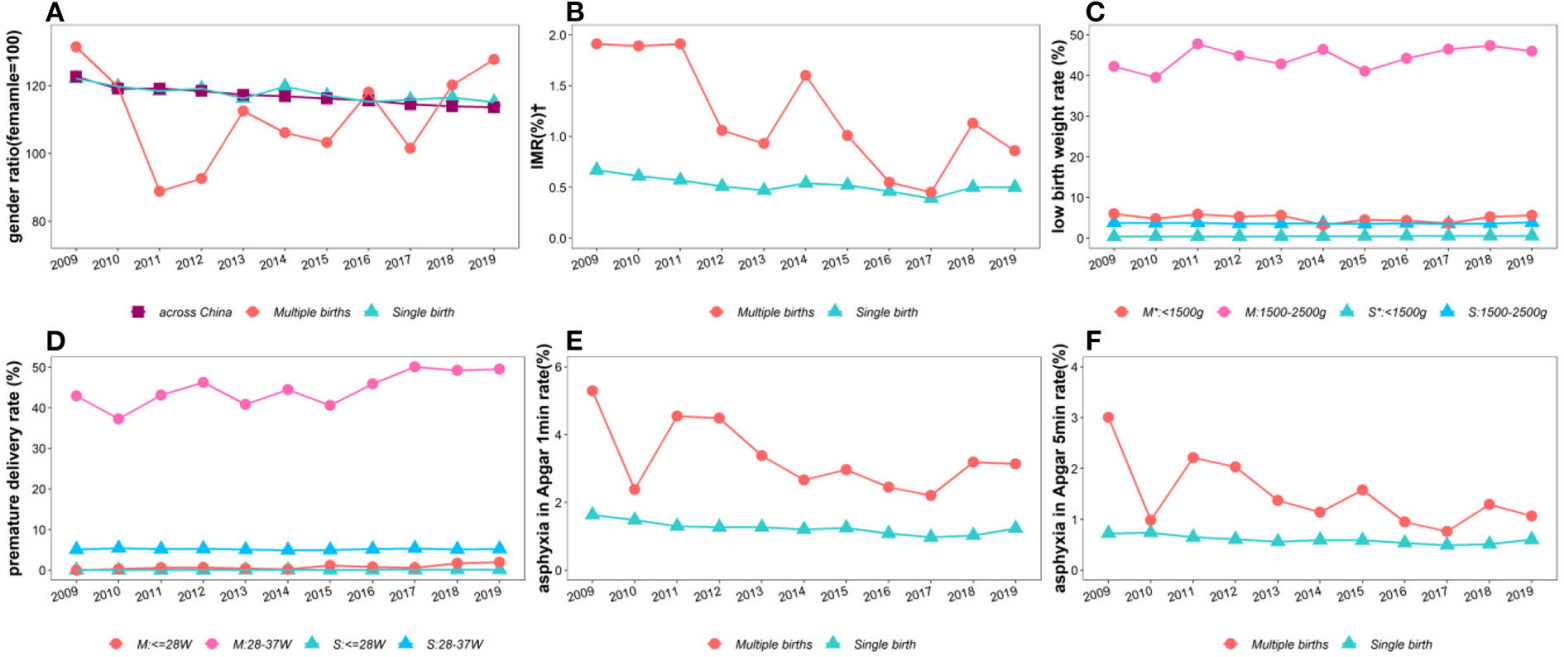
Secular trends of the demographic and developmental characteristics among 526,654 newborns in Baoan, Shenzhen, 2009–2019. **(A)** Gender ratio, **(B)** Infant Mortality Rate (IMR), **(C)** neonatal weight, **(D)** neonatal gestational age, **(E)** asphyxia rate with Apgar score <7 at 1 min after birth, **(F)** asphyxia rate with Apgar score <7 at 5 min after birth. *526,654 newborns includes 515,016 singletons and 11,638 multiple births. M: multiple births; S: single birth.^†^Infant mortality refers to the death within 0–7 days of birth.

The trends of neonatal sex ratio, mortality, low birth weight, premature birth and asphyxia over the past 11 years are also mapped in Figure 2 and Supplementary Figure S2. These trends and the significance of differences in slopes between groups were calculated using the same method as in the maternal trend analysis. The results showed that the sex ratio in Baoan, Shenzhen, was basically consistent with the national average level for the 0- to 4-year-old group (22), while this ratio in multiple births was lower than the national level in most years. The above indicators for singleton newborns have not changed significantly in the past 11 years, while the mortality rate (0–7 days) and hypoxia rate (Apgar score ≤7) of multiple fetuses have shown a downwards trend, with a declining rate over 50% since 2009. Multiple newborns with a birth weight of 1,500–2,500 g or a gestational age of 28–37 weeks accounted for more than 40%. This has increased continuously since 2015 and reached more than 50% in 2019. From the perspective of secular trends shown in Supplementary Figure S2, the slope changes of mortality and post-natal asphyxia rate of multiple births were significantly different from those of single births, while the changes of sex ratio, preterm birth rate, and VLBW rate were not significant.

### 3.3. Multivariate logistic regression analysis for the development level of multiple births

All multiple births in Baoan, Shenzhen, in 2009–2019 were used as the research object to evaluate the effects of maternal demographic characteristics, pregnancy-related complications and delivery on the fetal asphyxia rate (Apgar score) at 1 min and 5 min after birth and the very low body weight and intrauterine development (height/head circumference ratio). All of these variables were first analyzed by univariate analysis (Supplementary Tables S3–S6), with control variables added to account for random effects. Using the two levels of multiple births as the error source, a generalized linear mixed model (GLMM) was constructed for multivariate regression analysis. The results are shown in Table 3.

**Table 3:** Multivariate logistic analysis of multiple births in Baoan Shenzhen, 2009–2019.

	**Total (*N*, %)**	**Control (*N*, %)**	**Observation (*N*, %)**	**B**	**P**	**OR**
Apgar score at 1st min^*^^a^						
**Parity**						
1	3,126 (53.84)	2,996 (95.84)	130 (4.16)	Reference	–	–
2	2,041 (35.15)	1,968 (96.42)	73 (3.58)	−0.17	0.037	0.84 (0.72, 0.99)
3	529 (9.11)	505 (95.46)	24 (4.54)	−0.14	0.211	0.85 (0.67, 1.09)
≥4	102 (1.76)	95 (93.14)	7 (6.86)	−0.18	0.552	0.86 (0.53, 1.4)
**Antenatal care utilization rate**						
1	989 (17.03)	894 (90.39)	95 (9.61)	0.45	<0.001	1.57 (1.23, 2.02)
2	2,206 (38)	2,112 (95.74)	94 (4.26)	0.15	0.182	1.16 (0.93, 1.44)
3	969 (16.69)	944 (97.42)	25 (2.58)	Reference	–	–
4	1,642 (28.28)	1,622 (98.78)	20 (1.22)	−0.41	0.003	0.67 (0.51, 0.87)
**Delivery way** ^#^						
Cesarean section	4,846 (83.47)	4,731 (97.63)	115 (2.37)	Reference	–	–
Vaginal delivery	953 (16.41)	836 (87.72)	117 (12.28)	0.7	<0.001	2.01 (1.66, 2.43)
Apgar score at 5th min^*^^a^						
**Parity**						
1	3,125 (53.84)	3,078 (98.5)	47 (1.5)	Reference	–	–
2	2,040 (35.15)	2,021 (99.07)	19 (0.93)	−0.27	0.028	0.77 (0.6, 0.97)
3	529 (9.11)	520 (98.3)	9 (1.7)	−0.13	0.481	0.88 (0.62, 1.25)
≥4	102 (1.76)	101 (99.02)	1 (0.98)	−4.02	0.984	0.02 (0, inf)
**Antenatal care utilization rate**						
≤ 50%	987 (17.01)	955 (96.76)	32 (3.24)	0.55	0.006	1.73 (1.17, 2.57)
50–80%	2,206 (38.01)	2,173 (98.5)	33 (1.5)	0.34	0.064	1.4 (0.98, 1.99)
80–110%	969 (16.7)	963 (99.38)	6 (0.62)	Reference	–	–
>110%	1,642 (28.29)	1,637 (99.7)	5 (0.3)	−0.3	0.197	0.74 (0.47, 1.17)
**Delivery way** ^#^						
Cesarean section	4,845 (83.48)	4,809 (99.26)	36 (0.74)	Reference	–	–
Vaginal delivery	952 (16.4)	913 (95.9)	39 (4.1)	0.57	0	1.77 (1.35, 2.34)
VLBW^*^^b^						
**Parity** ^#^						
1	3,132 (53.83)	2,926 (93.42)	206 (6.58)	Reference	–	–
2	2,043 (35.12)	1,934 (94.66)	109 (5.34)	−0.2	0.007	0.82 (0.7, 0.95)
3	532 (9.14)	512 (96.24)	20 (3.76)	−0.58	<0.001	0.56 (0.42, 0.74)
≥4	103 (1.77)	96 (93.2)	7 (6.8)	−0.22	0.373	0.8 (0.5, 1.3)
**Antenatal care utilization rate**						
≤50%	994 (17.08)	857 (86.22)	137 (13.78)	0.65	<0.001	1.91 (1.49, 2.45)
50–80%	2,212 (38.02)	2,062 (93.22)	150 (6.78)	0.4	0.001	1.49 (1.19, 1.87)
80–110%	970 (16.67)	943 (97.22)	27 (2.78)	Reference	–	–
>110%	1,642 (28.22)	1,612 (98.17)	30 (1.83)	−0.19	0.173	0.83 (0.63, 1.09)
**High risk**						
No	2,237 (38.45)	2,056 (91.91)	181 (8.09)	Reference	–	–
Yes	3,581 (61.55)	3,418 (95.45)	163 (4.55)	−0.28	<0.001	0.75 (0.65, 0.87)
Height /head circumference ratio^*^^c^						
**Parity** ^#^						
1	3,113 (53.79)	2,894 (92.96)	219 (7.04)	Reference	–	–
2	2,036 (35.18)	1,920 (94.3)	116 (5.7)	−1.89	<0.001	0.15 (0.11, 0.21)
3	529 (9.14)	492 (93.01)	37 (6.99)	−1.82	<0.001	0.16 (0.11, 0.24)
≥4	101 (1.75)	96 (95.05)	5 (4.95)	−2.06	<0.001	0.13 (0.07, 0.22)
**Premature birth**						
No	3,128 (54.05)	3,008 (96.16)	120 (3.84)	Reference	–	–
Yes	2,659 (45.95)	2,402 (90.33)	257 (9.67)	0.41	<0.001	1.5 (1.34, 1.68)
**Hypertension**						
No	5,596 (96.7)	5,227 (93.41)	369 (6.59)	Reference	–	–
Yes	191 (3.3)	183 (95.81)	8 (4.19)	−0.35	0.039	0.7 (0.5, 0.98)
**Antenatal care utilization rate**					
≤ 50%	971 (16.78)	862 (88.77)	109 (11.23)	0.36	<0.001	1.44 (1.19, 1.74)
50–80%	2,208 (38.15)	2,060 (93.3)	148 (6.7)	0.12	0.15	1.13 (0.96, 1.32)
80–110%	966 (16.69)	919 (95.13)	47 (4.87)	Reference	–	–
>110%	1,642 (28.37)	1,569 (95.55)	73 (4.45)	−0.01	0.898	0.99 (0.82, 1.18)

The above multi-factor model make multiple births (twins vs. triplets) as random variables.

* Apgar score at 1st min: N_*Total*_ = 5,806, N_*Control*_ = 5,572, N_*Observation*_ = 234.

Apgar score at 5th min: N_*Total*_ = 5,804, N_*Control*_ = 5,728, N_*Observation*_ = 76.

VLBW: N_*Total*_ = 5,818, N_*Control*_ = 5,474, N_*Observation*_ = 344.

Height /head circumference ratio: N_*Total*_ = 5,787, N_*Control*_ = 5,410, N_*Observation*_ = 377.

# There are missing values.

a Adjust the effect of year, maternal age, premature birth, complication, education, high risk, hypertension, anemia, eclampsia, GBS infection, surgical indication.

b Adjust the effect of year, maternal age, surgical indication, complication, education, hypertension, anemia, eclampsia, GBS infection.

c Adjust the effect of year, maternal age, surgical indication, complication, education, high risk, anemia, eclampsia, GBS infection.

The independent risk factors for the Apgar score and its evaluation during the first minute of fetal delivery of a multiple pregnancy for hypoxia were parity (2nd vs. 1st, OR = 0.84) and natural birth (OR = 2.01). Compared to pre-natal care utilization rates of 80–110%, there was a 2.25-fold increased risk of neonatal hypoxia when the utilization rate was below 50%. When the utilization rate was >110%, it was a protective factor, and neonatal hypoxia was reduced by 51%. Independent risk factors for hypoxia at 5 min after birth were similar to those at 1 min after birth.

The independent influencing factors of VLBW in multiple birth newborns are the age of the parturient, parity, premature birth, pre-natal care utilization rate, and high-risk factors. The risk of VLBW in multiple birth fetuses decreased with increasing parity and the pregnant women's age but it was 5.11 times higher in premature fetuses than in full-term fetuses. Compared with standard pre-natal care (utilization rate 80–110%), the risk of VLBW increased 49% when the utilization rate was 50%-80%, increased 91% when the utilization rate was <50%, and decreased 17% when the utilization rate was >110%. When the mother had high-risk factors (hyperglycemia, maternal obesity, etc.) during pregnancy, the risk of VLBW was reduced. Cesarean section was a protective factor, which means that mothers with surgical indicators had a reduced risk of having a VLBW fetus.

According to the medical reference value of neonatal height and head circumference in China, we divided the height/head circumference ratio into two grades: normal (P10–P90) as the control group and abnormal (<P10 or >P90) as the observation group. These results showed that multiple newborns had a lower risk of an abnormal height/head circumference ratio than single infants; however, this risk of prematurity was higher than that of single infants (OR = 1.50). Compared with standard pre-natal care times (80–110%), the risk of a fetus falling into the abnormal ratio increased at a care utilization rate <50%. In addition, the risk of a low height-to-head circumference ratio was higher when the mother was educated for 6 years or less.

## 4. Discussion

Using the 2009–2019 maternal data collected from Baoan, Shenzhen, we investigated the primary trends in singleton and multiple births, the different characteristics between the two groups, and the factors affecting the development of multiple births. This long-term large retrospective study showed that the multiple birth rate has been increasing since 2014, while the mother's childbearing age has been significantly increasing. During this period, the cesarean section rate, the pre-natal care utilization rate, the application of assisted reproductive technology and the mother's education level have all increased. Simultaneously, pregnancy complications, fetal sex ratio, adverse outcomes and intrauterine adverse events have individually shown a decreasing trend. These changes reflect the improvement of reproductive health in Baoan, Shenzhen, which is related to the advances in medical care, the vigorous development of pregnant healthcare, the implementation of health security measures and the basic principles of attaching importance to education in China.

The characteristics of both singleton and multiple births changed periodically, but shown an upward or downward trend in a long time. Some characteristics in the multiple birth data show opposite trends to singleton data, such as birth rate, maternal complication rate, and percentage of ultra-light fetuses, all of which increase in multiple births and decrease in singleton births, and the rate of change (slope) is significant difference. There are also some characteristics that show an upward trend in both sets of data, such as maternal age, education level, preterm birth rate, pregnancy rate by assisted reproductive technology, pre-natal care utilization rate, etc., but multiple births trend to have larger slopes. The remaining characteristics show a downward trend in both groups of data, such as the vaginal delivery rate, the proportion of high-risk factors, the incidence of hypertension and eclampsia, the male-to-female ratio, etc., but the rate of decline exists significant differences. These results suggest that some indications of multiple births should be treated differently from singleton births in developing a long-term program.

Compared with mothers with singleton births, mothers with multiple births face higher reproductive risks, including higher rates of older maternity, preterm birth, and complications. Correspondingly, developmental indicators of multiple fetuses are universally inferior to those of singleton fetuses, which strongly suggests that during the pre-natal care of multiple birth mothers, the health monitoring and growth management of multiple fetuses should all be strengthened based on the standards of singletons.

Notably, the utilization rate of pre-natal care has an impact on the development of multiple births. When the utilization rate was <80%, the risk of very low birth weight, asphyxia or low height/head circumference in multiple births increased accordingly. Conversely, this risk clearly decreased when the utilization rate was >110%. This can be attributed to timely pre-natal examinations providing doctors with more accurate fetal development information, allowing for the adjustment of healthcare measures during pregnancy, giving women more scientific guidance, and improve the nutritional intake of pregnant women (23–25).

Premature infants, especially multiple births, are prone to hypoxia. This is consistent with the research showing that premature infants are prone to respiratory distress syndrome, which is caused by a lack of surfactant in fetal lungs. Pre-natal glucocorticoids (GCs) or ambroxol hydrochloride (HCL) have been well-documented to improve the synthesis of surfactant to promote lung maturation in human fetuses (26–28). When the mother suffers from pregnancy syndrome, the risk of fetal hypoxia increases. The results of our study are similar to those of a 5-year retrospective cohort study in Sweden (29). Perrine Lorain confirmed at the molecular level that the umbilical artery pH value of a hypoxic fetus is lower and the umbilical cord lactic acid level is higher (30).

The birth weight of a newborn is an important parameter to measure its health status. Multiple birth fetuses are more likely to be underweight than singleton fetuses. We found that multiparity is a protective factor against very low fetal birth weight. Several studies (31–33) have found that the fetal birth weight increases with the number of births of a mother. Johnson et al. (34) believed that the increase in birth weight by birth number was affected by the gradual increase in uterine blood flow, which is related to the weight gain of the offspring. Olle Hartvigsson et al. (35) also proved this by plasma metabolomics of umbilical cords in arteries and veins.

The recommended pre-natal care number for pregnant women in China is 10 times (4 ultrasounds included) (36), and there is no difference in the recommendation between singletons and multiple births. The WHO has suggested that every pregnant woman should receive at least eight visits before giving birth (17). From the data presented here, it is clear that there may be a need to update the pre-natal care standard for multiple births, such as by increasing the frequency of ultrasonography to expose potential risks earlier. After changing the population policy in China, the proportion of multiple births among newborns is on the rise, which requires current maternity care methods to keep up with this trend.

Since the initiation of the two-child policy in 2015, the use of assisted reproductive technology has increased rapidly, resulting in more multiple births. In 2022, China revised the Population and Family Planning Law to promote the implementation of the marriageable age, eugenics and three-child policy. With the removal of birth restrictions, the use of assisted reproductive technology may be more extensive. However, there is currently a lack of comprehensive regulations to limit its extent, including the number of implanted embryos, and the possibility of sex selection, which may lead to greater physical burdens for women (37). With the application of assisted reproductive technology, human genetic material no longer undergoes natural selection during reproduction. How to control resource spillover and avoid unnecessary economic losses while giving full play to the therapeutic effect of this technology on infertile families is a question worth considering. Finally, how to reduce the risks of stillbirth, miscarriage and deformity during the process of implementing this technology is also an urgent problem.

## 5. Limitation

This is a retrospective cohort study that did not subdivide the high-risk factors and syndrome subgroups of pregnant women.

## Data availability statement

The original contributions presented in the study are included in the article/Supplementary material, further inquiries can be directed to the corresponding author.

## Ethics statement

The studies involving human participants were reviewed and approved by Medical Ethics Committee of Shenzhen Baoan Women's and Children's Hospital, Jinan University. Written informed consent for participation was not required for this study in accordance with the national legislation and the institutional requirements.

## Author contributions

LZ conceived and designed the study, reviewed the draft, provided oversight and expert advice for the research, and the written paper. WT and LZ collected the data, performed data analysis, and drafted the manuscript. All authors reviewed subsequent drafts and approved the final version of the paper.

## References

[1] Martin JA Hamilton BE Osterman MJ. Three decades of twin births in the United States, 1980-2009. NCHS Data Brief . (2012) 80:1–8. 22617378

[2] Committeeon Practice Bulletins Obstetrics and the Society for Materna Fetal Medicine. Multifetal Gestations: twin, triplet, and higher-order multifetal pregnancies. ACOG practice bulletin, number 231. Obstetr Gynecol. (2021) 137:e145–62. 10.1097/AOG.0000000000004397 34011891

[3] Wang L Dongarwar D Salihu HM. Temporal trends in the rates of singletons, twins and higher-order multiple births over five decades across racial groups in the United States. Int J MCH AIDS. (2020) 9:257–9. 10.21106/ijma.377 32724725 PMC7376828

[4] Martin JA Hamilton BE Osterman MJK Driscoll AK. Births: final data for 2018. Natl Vital Stat Rev. (2019) 68:1–47. 32501202

[5] Aniket DK Denise JJ Howard WJ Dmitry MK Maria FG Maurizio M. Fertility treatments and multiple births in the United States. N Engl J Med. (2013) 369:2218–25. 10.1056/NEJMoa1301467 24304051

[6] Xinrong L Jun Z Yinghui L Ting W Yanyu L Zhu L. Epidemiology of twin births in southeast China: 1993–2005. Twin Res Hum Genet. (2013) 16:608–13. 10.1017/thg.2013.7 23425723

[7] Han E Song J Liu A Huo K Xu F Cui S. Trends in live births in the past 20 years in Zhengzhou, China. Acta Obstet Gynecol Scand. (2011) 90:332–7. 10.1111/j.1600-0412.2010.01065.x 21306327

[8] Laura AS Susan FM Cynthia F Herbert BP Gary J Lynne SW. Low and very low birth weight in infants conceived with use of assisted reproductive technology. N Engl J Med. (2002) 346:731–7. 10.1056/NEJMoa010806 11882728

[9] Prakesh SS. Parity and low birth weight and preterm birth: a systematic review and meta-analyses. Acta Obstet Gynecol Scand. (2010) 89:862–75. 10.3109/00016349.2010.486827 20583931

[10] Jie H Hongzhi Z Joseph MB Tongzhang Z Bin Z Wei X. Associations of trimester-specific exposure to bisphenols with size at birth: a Chinese pre-natal cohort study. Environ Health Perspect. (2019) 127:107001. 10.1289/EHP4664 31573832 PMC6867404

[11] Chang C Jinwen Z Hongwei X Huixin Z Ana PB Lin Z. Preterm birth in China between 2015 and 2016. Am J Public Health. (2019) 109:1597–604. 10.2105/AJPH.2019.305287 31536409 PMC6775901

[12] World Health Organization. Monitoring Childbirth in a New Era for Maternal Health. (2021). Available online at: https://www.who.int/news/item/15-12-2020-monitoring-childbirth-in-a-new-era-for-maternal-health (accessed June 8, 2022).

[13] National Health Health. Notice of the National Health and Health Commission on Implementing the Decision of the CPC Central Committee and the State Council on Optimizing the Birth Policy and Promoting Long-term Balanced Population Development. (2021). Available online at: http://www.nhc.gov.cn/rkjcyjtfzs/s7786/202107/8b54cdbc38b5440a876734fc2ba08f72.shtml (accessed June 19, 2022).

[14] Rui M Lingyun Z. Stillbirth trends by maternal sociodemographic characteristics among a large internal migrant population in Shenzhen, China, over a 10-year period: a retrospective study. BMC Public Health. (2022) 22:325. 10.1186/s12889-022-12734-8 35172785 PMC8848954

[15] Zhijiang L Yan L Yuanzhu M Lei Z Xue Z Li L. The association between ambient temperature and preterm birth in Shenzhen, China: a distributed lag non-linear time series analysis. Environ Health. (2016) 15:84. 10.1186/s12940-016-0166-4 27503307 PMC4977688

[16] Xinnan Z Hui L Yaqin Z Huahong W. Growth reference standards for weight / head circumference ratio and length / head circumference ratio of newborns in China. Chin J Evid Based Pediatr. (2020) 15:401–5. 10.3969/j.issn.1673-5501.2020.06.001

[17] World Health Organization. WHO Recommendations on Antenatal Care for a Positive Pregnancy Experience. World Health Organization (2016). Available online at: https://www.who.int/publications/i/item/9789241549912 28079998

[18] Hong L Feiyang D Jiangbo D Ting C Qingxia M Xiufeng L. Assisted reproductive technology and birth defects in a Chinese birth cohort study. Lancet Reg Health West Pac. (2021) 7:100090. 10.1016/j.lanwpc.2020.100090 34327418 PMC8315325

[19] Christine F Yujia Z Dmitry MK Laura AS Denise JJ Catherine R. Association between assisted reproductive technology conception and autism in California, 1997–2007. Am J Public Health. (2015) 105:963–71. 10.2105/AJPH.2014.302383 25790396 PMC4386543

[20] Rebecca FL Kelly DG Dominique H Barbara L Judy ES Eugene RD. Assisted reproductive technology and birth defects: effects of subfertility and multiple births. Birth Defects Res. (2017) 109:1144–53. 10.1002/bdr2.1055 28635008 PMC5555800

[21] Wen T Lingmin H Juan W. Maternal and neonatal outcomes after assisted reproductive technology: a retrospective cohort study in China. Front Med. (2022) 9:837762. 10.3389/fmed.2022.837762 35479950 PMC9037083

[22] Bureau of statistics of the people's Republic of China. China Statistical Yearbook (2010–2020). Beijing: China Statistics Press (2020).

[23] Jennifer CR Yunxi Z Dorthy KY Xiaojian L Elizabeth AL. Use of telehealth services for pre-natal care in mississippi: comparison of pre-COVID-19 pandemic and pandemic obstetric management. Int J Clin Pract. (2022) 2022:3535700. 10.1155/2022/3535700 35685499 PMC9159141

[24] Alqurashi MA. Survival rate of very low birth weight infants over a quarter century (1994–2019): a single-institution experience. J Neonatal Perinatal Med. (2021) 14:253–60. 10.3233/NPM-200595 33074199

[25] Cuestas E Gomez-Flores ME Charras MD Peyrano AJ Montenegro C Sosa-Boye I. Socioeconomic inequalities in low birth weight risk before and during the COVID-19 pandemic in Argentina: a cross-sectional study. Lancet Reg Health Am. (2021) 2:100049. 10.1016/j.lana.2021.100049 34642686 PMC8495179

[26] Wynne K Rowe C Delbridge M Watkins B Brown K Addley J. Antenatal corticosteroid administration for foetal lung maturation. F1000Research. (2020) 9:219. 10.12688/f1000research.20550.1 32269758 PMC7111495

[27] Backes CH Rivera BK Pavlek L Beer LJ Ball MK Zettler ET. Proactive neonatal treatment at 22 weeks of gestation: a systematic review and meta-analysis. Am J Obstet Gynecol. (2021) 224:158–74. 10.1016/j.ajog.2020.07.051 32745459

[28] Ehret DEY Edwards EM Greenberg LT Bernstein IM Buzas JS Soll RF. Association of antenatal steroid exposure with survival among infants receiving postnatal life support at 22 to 25 weeks' gestation. JAMA Netw Open. (2018) 1:e183235. 10.1001/jamanetworkopen.2018.3235 30646235 PMC6324435

[29] Cecilia L Lars B Anna-Stina W Marie B. Ante- and intrapartum risk factors for neonatal hypoxic ischemic encephalopathy. J Matern Fetal Neonatal Med. (2018) 31:1595–601. 10.1080/14767058.2017.1321628 28486858

[30] Lorain P Bower A Gottardi E Dommergues M Foix L'Helias L Guellec I. Risk factors for hypoxic-ischemic encephalopathy in cases of severe acidosis: a case-control study. Acta Obstet Gynecol Scand. (2022) 101:471–8. 10.1111/aogs.14326 35338480 PMC9564668

[31] Camill AP Cremon V. The effect of parity on birthweight. J Obstetr Gynaecol Br Commonwealth. (1970) 77:145–7. 10.1111/j.1471-0528.1970.tb03493.x 5419878

[32] Muula AS Siziya S Rudatsikira E. Parity and maternal education are associated with low birth weight in Malawi. Afr Health Sci. (2011) 11:65–71. 21572859 PMC3092318

[33] Overland EA Vatten LJ Eskild A. Risk of shoulder dystocia: associations with parity and offspring birthweight. A population study of 1 914 544 deliveries. Acta Obstet Gynecol Scand. (2012) 91:483–8. 10.1111/j.1600-0412.2011.01354.x 22356510

[34] Mark AW Allan MZC Ian RJ. The effects of parity on birthweight using successive pregnancies. Acta Obstetr Gynecol Scand. (1996) 75:459–3. 10.3109/00016349609033354 8677771

[35] Hartvigsson O Barman M Savolainen O Ross AB Sandin A Jacobsson B. Differences between arterial and venous umbilical cord plasma metabolome and association with parity. Metabolites. (2022) 12:175. 10.3390/metabo12020175 35208249 PMC8877791

[36] Obstetrics Subgroup & Chinese Society of Obstetrics Gynecology & Chinese Medical Association. Guideline of preconception and prenatal care 2018. Chin J Obstet Gynecol. (2018) 53:7–13. 10.3760/cma.j.issn.0529-567X.2018.01.003 29374879

[37] Nerissa W Thomas H Olaf P Joel T Robert H Ellen H. Human exposure to PBDEs: associations of PBDE body burdens with food consumption and house dust concentrations. Environ Sci Technol. (2007) 41:1584–9. 10.1021/es0620282 17396645

