# Supplementary material for: Trends and characteristics of multiple births in Baoan Shenzhen: A retrospective study over a decade
Source: Front Public Health. 2022 Dec 13;10:1025867. doi: 10.3389/fpubh.2022.1025867 (PMC9793989; doi:10.3389/fpubh.2022.1025867)
Supplement: Supplementary file 1 [file Data_Sheet_1.PDF]

Supplementary Material

1 Supplementary Tables

Table S1. Descriptive statistics for mothers with multiple and single births in Baoan Shenzhen, 2009-2019

	Total	single birth	multiple births	P[2]
	(N=520860[1], %)	(N=515016[1],%)	(N=5844[1],%)	
**Year of delivery**				<0.001
**2009**	37809(7.26)	37451(7.27)	358(6.13)	
**2010**	41039(7.88)	40681(7.9)	358(6.13)	
**2011**	45747(8.78)	45338(8.8)	409(7)	
**2012**	54243(10.41)	53773(10.44)	470(8.04)	
**2013**	46315(8.89)	45842(8.9)	473(8.09)	
**2014**	49554(9.51)	49027(9.52)	527(9.02)	
**2015**	45300(8.7)	44759(8.69)	541(9.26)	
**2016**	50539(9.7)	49905(9.69)	634(10.85)	
**2017**	52121(10.01)	51462(9.99)	659(11.28)	
**2018**	48433(9.3)	47773(9.28)	660(11.29)	
**2019**	49760(9.55)	49005(9.52)	755(12.92)	
**Two child policy**				<0.001
**no**	340680(65.41)	337275(65.49)	3405(58.26)	
**yes**	180180(34.59)	177741(34.51)	2439(41.74)	
**Maternal age (years)**				<0.001
**Mean (SD)**	28.3 (4.89)	28.3 (4.89)	30.1 (5.11)	
**Median [Min, Max]**	28.0 [15.0, 58.0]	28.0 [15.0, 58.0]	30.0 [15.0, 53.0]	
**Missing**	198(0.04)	198(0.04)	0	
**Maternal age**				<0.001
**＜19**	6012(1.15)	5982(1.16)	30(0.51)	
**19~23**	54586(10.48)	54244(10.53)	342(5.85)	

**23~35**	402683(77.31)	398267(77.33)	4416(75.56)	
**35~40**	48649(9.34)	47809(9.28)	840(14.37)	
**≥40**	8732(1.68)	8516(1.65)	216(3.7)	
**Missing**	198(0.04)	198(0.04)	0	
**Parity**				<0.001
**1**	241025(46.27)	237877(46.19)	3148(53.87)	
**2**	226954(43.57)	224897(43.67)	2057(35.2)	
**3**	44410(8.53)	43880(8.52)	530(9.07)	
**≥4**	6333(1.22)	6249(1.21)	84(1.44)	
**Missing**	2138(0.41)	2113(0.41)	25(0.43)	
**Hypertension**				<0.001
**no**	498414(95.69)	493297(95.78)	5117(87.56)	
**yes**	22446(4.31)	21719(4.22)	727(12.44)	
**Education degree (yesrs)**				<0.001
**≤6**	10286(1.97)	10179(1.98)	107(1.83)	
**6~12**	371525(71.33)	367649(71.39)	3876(66.32)	
**＞12**	139049(26.7)	137188(26.64)	1861(31.84)	
**Reproductive Techniques**				<0.001
**no**	519823(99.8)	514330(99.87)	5493(93.99)	
**yes**	1037(0.2)	686(0.13)	351(6.01)	
**Prenatal care utilization rate**				<0.001
**≤50%**	108532(20.84)	107550(20.88)	982(16.8)	
**50-80%**	216743(41.61)	214517(41.65)	2226(38.09)	
**80-110%**	70629(13.56)	69654(13.52)	975(16.68)	
**＞110%**	124888(23.98)	123229(23.93)	1659(28.39)	
**Missing**	68(0.01)	66(0.01)	2(0.03)	
**Surgical indication**				<0.001
**no**	330895(63.53)	329868(64.05)	1027(17.57)	
**yes**	189965(36.47)	185148(35.95)	4817(82.43)	

**Complication**				<0.001
**no**	442014(84.86)	438355(85.11)	3659(62.61)	
**yes**	78846(15.14)	76661(14.89)	2185(37.39)	
**Eclampsia**				<0.001
**no**	514840(98.84)	509352(98.9)	5488(93.91)	
**yes**	6020(1.16)	5664(1.1)	356(6.09)	
**GBS infection**				0.400
**no**	519821(99.8)	513986(99.8)	5835(99.85)	
**yes**	1039(0.2)	1030(0.2)	9(0.15)	
**Anemia**				<0.001
**no**	516039(99.07)	510282(99.08)	5757(98.51)	
**yes**	4821(0.93)	4734(0.92)	87(1.49)	
**High risk**				<0.001
**no**	352621(67.7)	351158(68.18)	1463(25.03)	
**yes**	168239(32.3)	163858(31.82)	4381(74.97)	
**Emergency**				<0.001
**no**	519822(99.8)	514027(99.81)	5795(99.16)	
**yes**	1038(0.2)	989(0.19)	49(0.84)	
**Pregnancy week**				<0.001
**Mean (SD)**	38.8 (1.66)	38.9 (1.62)	36.1 (2.35)	
**Median [Min, Max]**	39.0 [24.0, 50.0]	39.0 [24.0, 50.0]	37.0 [24.0, 43.0]	
**Pregnancy week**				<0.001
**<28**	317(0.06)	266(0.05)	51(0.87)	
**28-37**	29213(5.61)	26564(5.16)	2649(45.33)	
**37-42**	485797(93.27)	482660(93.72)	3137(53.68)	
**≥42**	5533(1.06)	5526(1.07)	7(0.12)	
**Delivery mode**				<0.001
**caesarean section**	172971(33.21)	168098(32.64)	4873(83.38)	
**vaginal delivery**	347772(66.77)	346808(67.34)	964(16.5)	
**else**	117(0.02)	110(0.02)	7(0.12)	

**Postpartum hemorrhage**					<0.001
	**no**	515000(98.87)	509395(98.91)	5605(95.91)	
	**yes**	5855(1.12)	5616(1.09)	239(4.09)	
	**Missing**	5	5	0	
**Maternal outcome**					<0.001
	**no**	520815(99.99)	514972(99.99)	5843(99.98)	
	**yes**	45(0.01)	44(0.01)	1(0.02)	
**Smoking**					>0.9
	**no**	98.7 (12.3)	98.9 (12.1)	77.7 (12.7)	
	**yes**	100 [18.9, 395]	100 [18.9, 395]	79.0 [26.3, 173]	
	**Missing**	93218(17.9)	91967(17.86)	1251(21.41)	
**Drinking**					>0.9
	**no**	427555(82.09)	422962(82.13)	4593(78.59)	
	**yes**	90(0.02)	90(0.02)	0	
	**Missing**	93215(17.9)	91964(17.86)	1251(21.41)	
**Husband smoking**					0.200
	**no**	70056(13.45)	69420(13.48)	636(10.88)	
	**yes**	60259(11.57)	59748(11.6)	511(8.74)	
	**Missing**	390545(74.98)	385848(74.92)	4697(80.37)	
**Husband drinking**					0.700
	**no**	79812(15.32)	79095(15.36)	717(12.27)	
	**yes**	44334(8.51)	43945(8.53)	389(6.66)	
	**Missing**	396714(76.17)	391976(76.11)	4738(81.07)	
**Bad pregnancy and childbirth history**					0.200
	**no**	461650(88.63)	456504(88.64)	5146(88.06)	
	**yes**	59210(11.37)	58512(11.36)	698(11.94)	
**Referral from grass-roots level**					>0.9
	**no**	520793(99.99)	514949(99.99)	5844(100)	
	**yes**	67(0.01)	67(0.01)	0	

1 n (%); Median (IQR)
2 Pearson's Chi-squared test; Wilcoxon rank sum test; Fisher's exact test

Table S2. Descriptive statistics for newborns with multiple and single births in Baoan Shenzhen, 2009-2019

	Total	single birth	multiple births	P[2]
	(N=526654[1],%)	(N=515016[1],%)	(N=11638[1],%)	
**Year of delivery**				<0.001
**2009**	38150(7.24)	37451(7.27)	699(6.01)	
**2010**	41395(7.86)	40681(7.9)	714(6.14)	
**2011**	46152(8.76)	45338(8.8)	814(6.99)	
**2012**	54709(10.39)	53773(10.44)	936(8.04)	
**2013**	46790(8.88)	45842(8.9)	948(8.15)	
**2014**	50078(9.51)	49027(9.52)	1051(9.03)	
**2015**	45838(8.7)	44759(8.69)	1079(9.27)	
**2016**	51170(9.72)	49905(9.69)	1265(10.87)	
**2017**	52776(10.02)	51462(9.99)	1314(11.29)	
**2018**	49090(9.32)	47773(9.28)	1317(11.32)	
**2019**	50506(9.59)	49005(9.52)	1501(12.9)	
**Two child policy**				<0.001
**no**	344054(65.33)	337275(65.49)	6779(58.25)	
**yes**	182600(34.67)	177741(34.51)	4859(41.75)	
**Pregnancy week**				<0.001
**Mean (SD)**	38.8 (1.69)	38.9 (1.62)	36.1 (2.34)	
**Median [Min, Max]**	39.0 [24.0, 50.0]	39.0 [24.0, 50.0]	37.0 [24.0, 43.0]	
**Pregnancy week**				<0.001
**<28**	366(0.07)	266(0.05)	100(0.86)	
**28-37**	31835(6.04)	26564(5.16)	5271(45.29)	
**37-42**	488914(92.83)	482660(93.72)	6254(53.74)	
**≥42**	5539(1.05)	5526(1.07)	13(0.11)	

**Gender**				0.002
**female**	242173(45.98)	236653(45.95)	5520(47.43)	
**male**	284471(54.01)	278353(54.05)	6118(52.57)	
**Missing**	10	10	0	
**Position**				<0.001
**LOA**	437871(83.14)	429077(83.31)	8794(75.56)	
**LSA**	12650(2.4)	11711(2.27)	939(8.07)	
**ROA**	20299(3.85)	20000(3.88)	299(2.57)	
**RSA**	1164(0.22)	985(0.19)	179(1.54)	
**Tire**	4008(0.76)	3890(0.76)	118(1.01)	
**Else**	31295(5.94)	30552(5.93)	743(6.38)	
**Missing**	19367(3.68)	18801(3.65)	566(4.86)	
**Baby outcome**				<0.001
**born alive**	524388(99.57)	512835(99.58)	11553(99.27)	
**early neonatal death**	223(0.04)	206(0.04)	17(0.15)	
**stillbirth**	1703(0.32)	1651(0.32)	52(0.45)	
**Missing**	340(0.06)	324(0.06)	16(0.14)	
**Malformation**				<0.001
**no**	516504(98.07)	505299(98.11)	11205(96.28)	
**yes**	10150(1.93)	9717(1.89)	433(3.72)	
**Apgar score at 1 min**				0.5
**Mean (SD)**	9.71 (0.861)	9.71 (0.854)	9.61 (1.15)	
**Median [Min, Max]**	10.0 [0, 10.0]	10.0 [0, 10.0]	10.0 [0, 10.0]	
**Apgar score at 1 min**				<0.001
**0~3**	2590(0.49)	2483(0.48)	107(0.92)	
**4~7**	4123(0.78)	3856(0.75)	267(2.29)	
**8~10**	519941(98.73)	508677(98.77)	11264(96.79)	

**Apgar score at 5 min**					<0.001
	**Mean (SD)**	9.93 (0.677)	9.93 (0.671)	9.85 (0.902)	
	**Median [Min, Max]**	10.0 [0, 10.0]	10.0 [0, 10.0]	10.0 [0, 10.0]	
**Apgar score at 5 min**					<0.001
	**0~3**	2194(0.42)	2115(0.41)	79(0.68)	
	**4~7**	1025(0.19)	942(0.18)	83(0.71)	
	**8~10**	523435(99.39)	511959(99.41)	11476(98.61)	
**Weight(g)**					<0.001
	**Mean (SD)**	3220 (477)	3230 (461)	2420 (493)	
	**Median [Min, Max]**	3200 [500, 6850]	3250 [500, 6850]	2500 [500, 4280]	
**Weight(g)**					<0.001
**<28**	**Mean (SD)**	927.26(193.07)	946.98(198.29)	874.8(168.42)	
	**Median [Q1,Q3]**	912.5[800,1050]	950[800,1080]	880[745,990]	
**28~37**	**Mean (SD)**	2349.12(559.88)	2384.12(569.83)	2172.73(468.62)	
	**Median [Q1,Q3]**	2400[2000,2720]	2450[2000,2800]	2200[1890,2500]	
**37~42**	**Mean (SD)**	3272.27(407.53)	3280.26(402.1)	2655.63(346.49)	
	**Median [Q1,Q3]**	3250[3000,3500]	3260[3000,3500]	2650[2420,2900]	
**≥42**	**Mean (SD)**	3466.9(438.88)	3468.24(438.19)	2896.92(364.04)	
	**Median [Q1,Q3]**	3450[3200,3750]	3450[3200,3750]	2860[2600,3050]	
**Weight(g)**					<0.001
	**<1500**	2965(0.56)	2399(0.47)	566(4.86)	
	**1500~2500**	23994(4.56)	18788(3.65)	5206(44.73)	
	**2500~4000**	474433(90.08)	468570(90.98)	5863(50.38)	
	**>=4000**	25262(4.8)	25259(4.9)	3(0.03)	
**Height(cm)**					<0.001
	**Mean (SD)**	49.8 (1.89)	49.9 (1.77)	46.6 (3.37)	

	**Median [Min, Max]**	50.0 [25.0, 65.0]	50.0 [25.0, 65.0]	48.0 [25.0, 54.0]	
**Height(cm)**					<0.001
**<28**	**Mean (SD)**	34.04(3.09)	34.33(3.24)	33.29(2.5)	
	**Median [Q1,Q3]**	34[32,36]	35[32,36]	33[31,35]	
**28~37**	**Mean (SD)**	45.99(3.85)	46.16(3.89)	45.11(3.56)	
	**Median [Q1,Q3]**	47[44,49]	47[45,49]	46[43,48]	
**37~42**	**Mean (SD)**	50.06(1.27)	50.09(1.25)	48.11(1.84)	
	**Median [Q1,Q3]**	50[50,51]	50[50,51]	48[47,49]	
**≥42**	**Mean (SD)**	50.49(1.4)	50.49(1.4)	49.08(1.26)	
	**Median [Q1,Q3]**	50[50,51]	50[50,51]	49[48,50]	
**Head circumference(cm)**					<0.001
	**Mean (SD)**	33.7 (1.28)	33.8 (1.24)	32.1 (1.98)	
	**Median [Min, Max]**	34.0 [15.0, 50.0]	34.0 [15.0, 50.0]	33.0 [15.0, 39.0]	
**Head circumference(cm)**					<0.001
**<28**	**Mean (SD)**	24.72(2.59)	24.73(2.61)	24.67(2.57)	
	**Median [Q1,Q3]**	25[23,26]	25[23,26]	25[23,26]	
**28~37**	**Mean (SD)**	31.52(2.34)	31.56(2.38)	31.34(2.13)	
	**Median [Q1,Q3]**	32[30,33]	32[30,33]	32[30,33]	
**37~42**	**Mean (SD)**	33.88(1)	33.89(0.99)	32.83(1.19)	
	**Median [Q1,Q3]**	34[33,34]	34[33,34]	33[32,34]	
**≥42**	**Mean (SD)**	34.12(1.1)	34.12(1.1)	33.77(0.93)	
	**Median [Q1,Q3]**	34[34,35]	34[34,35]	34[33,34]	
**Chest circumference(cm)**					<0.001

	Mean (SD)	32.6 (1.43)	32.6 (1.39)	30.9 (2.17)	
	Median [Min, Max]	33.0 [10.0, 60.0]	33.0 [10.0, 60.0]	32.0 [10.0, 50.0]	
**Chest circumference(cm)**					
**<28**	**Mean (SD)**	23.39(2.84)	23.41(2.7)	23.31(3.2)	
	**Median [Q1,Q3]**	23[22,25]	23[22,25]	23.5[22,25]	
**28~37**	**Mean (SD)**	30.31(2.51)	30.35(2.54)	30.11(2.35)	
	**Median [Q1,Q3]**	31[29,32]	31[29,32]	31[29,32]	
**37~42**	**Mean (SD)**	32.74(1.16)	32.76(1.15)	31.73(1.33)	
	**Median [Q1,Q3]**	33[32,33]	33[32,33]	32[31,33]	
**≥42**	**Mean (SD)**	32.88(1.36)	32.88(1.36)	32.62(1.5)	
	**Median [Q1,Q3]**	33[32,34]	33[32,34]	32[32,34]	
**Height/Head circumference**					<0.001
	**Mean (SD)**	1.48 (0.0410)	1.48 (0.0398)	1.45 (0.0711)	
	**Median [Min, Max]**	1.47 [0.660, 3.33]	1.47 [0.660, 2.76]	1.45 [0.910, 3.33]	
**Height/Head circumference**					<0.001
**<28**	**Mean (SD)**	1.39(0.13)	1.4(0.14)	1.36(0.12)	
	**Median [Q1,Q3]**	1.38[1.32,1.46]	1.4[1.32,1.48]	1.36[1.295,1.43]	
**28~37**	**Mean (SD)**	1.46(0.08)	1.46(0.08)	1.44(0.09)	
	**Median [Q1,Q3]**	1.47[1.43,1.5]	1.47[1.44,1.5]	1.45[1.39,1.48]	
**37~42**	**Mean (SD)**	1.48(0.04)	1.48(0.04)	1.46(0.05)	
	**Median [Q1,Q3]**	1.47[1.46,1.5]	1.47[1.46,1.5]	1.47[1.44,1.48]	
**≥42**	**Mean (SD)**	1.48(0.04)	1.48(0.04)	1.45(0.05)	
	**Median [Q1,Q3]**	1.47[1.46,1.5]	1.47[1.46,1.5]	1.45[1.44,1.5]	
**Weight/Head circumference**					<0.001

	Mean (SD)	95.1 (12.0)	95.5 (11.6)	75.0 (12.6)	
	Median [Min, Max]	95.6 [17.9, 180]	95.8 [17.9, 180]	76.4 [20.8, 118]	
**Weight/Head circumference**					<0.001
**<28**	**Mean (SD)**	37.43(6.19)	38.2(6.35)	35.35(5.23)	
	**Median [Q1,Q3]**	37.45[33.26,41.48]	38.33[33.83,42.07]	35.58[31.14,39.025]	
**28~37**	**Mean (SD)**	73.82(14.14)	74.81(14.31)	68.83(12.08)	
	**Median [Q1,Q3]**	75.15[65.38,83.33]	76.36[66.67,84.85]	70[61.29,77.42]	
**37~42**	**Mean (SD)**	96.43(10.32)	96.63(10.18)	80.75(9.1)	
	**Median [Q1,Q3]**	96.97[89.39,102.94]	96.97[89.71,102.94]	80.59[75,86.76]	
**≥42**	**Mean (SD)**	101.47(11.09)	101.5(11.07)	85.77(10.3)	
	**Median [Q1,Q3]**	100[94.12,108.57]	100.28[94.12,108.57]	83.82[79.41,92.42]	
**Height/Chest circumference**					<0.001
	**Mean (SD)**	1.53 (0.0558)	1.53 (0.0550)	1.51 (0.0812)	
	**Median [Min, Max]**	1.52 [0.630, 5.10]	1.52 [0.630, 5.10]	1.52 [0.970, 3.57]	
**Height/Chest circumference**					<0.001
**<28**	**Mean (SD)**	1.47(0.16)	1.48(0.16)	1.45(0.17)	
	**Median [Q1,Q3]**	1.46[1.38,1.57]	1.48[1.38,1.57]	1.43[1.355,1.555]	
**28~37**	**Mean (SD)**	1.52(0.09)	1.52(0.09)	1.5(0.1)	
	**Median [Q1,Q3]**	1.52[1.48,1.56]	1.52[1.48,1.56]	1.5[1.45,1.55]	
**37~42**	**Mean (SD)**	1.53(0.05)	1.53(0.05)	1.52(0.06)	
	**Median [Q1,Q3]**	1.52[1.5,1.56]	1.52[1.5,1.56]	1.52[1.48,1.55]	
**≥42**	**Mean (SD)**	1.54(0.07)	1.54(0.07)	1.51(0.06)	
	**Median [Q1,Q3]**	1.53[1.51,1.56]	1.53[1.51,1.56]	1.5[1.46,1.55]	

**Weight/Chest circumference**					<0.001
	**Mean (SD)**	98.4 (12.5)	98.9 (12.1)	77.8 (12.9)	
	**Median [Min, Max]**	99.4 [18.9, 395]	100 [18.9, 395]	79.0 [22.7, 173]	
**Weight/Chest circumference**					<0.001
**<28**	**Mean (SD)**	39.67(6.78)	40.44(6.95)	37.62(5.85)	
	**Median [Q1,Q3]**	39.2[35,43.75]	40.4[35.42,45.2]	37.5[33.265,41.55]	
**28~37**	**Mean (SD)**	76.76(14.51)	77.77(14.69)	71.64(12.39)	
	**Median [Q1,Q3]**	78.13[67.93,86.67]	79.17[68.97,87.5]	72.81[64.14,80.3]	
**37~42**	**Mean (SD)**	99.8(10.84)	100.01(10.7)	83.57(9.41)	
	**Median [Q1,Q3]**	100[92.42,106.25]	100[93.33,106.25]	83.33[77.5,90]	
**≥42**	**Mean (SD)**	105.34(11.68)	105.38(11.65)	88.8(9.85)	
	**Median [Q1,Q3]**	105.71[96.97,112.5]	105.88[96.97,112.5]	89.06[84.12,96.77]	

1 n (%); Median (IQR)/(Q1,Q3)
2 Pearson's Chi-squared test; Wilcoxon rank sum test; Fisher's exact test

Table S3. Difference analysis for multiple births with birth at 1 min apgar score in Baoan Shenzhen, 2009-2019

		Total	non-asphyxiation	Asphyxiation	p*
		**(N=5806,%)**	**(N=5572,%)**	**(N=234,%)**	
**Maternal age**					0.1
	**＜19**	30(0.52)	27(0.48)	3(1.28)	
	**19~23**	208(3.58)	196(3.52)	12(5.13)	
	**23~35**	4513(77.73)	4341(77.91)	172(73.5)	
	**35~40**	839(14.45)	805(14.45)	34(14.53)	
	**≥40**	216(3.72)	203(3.64)	13(5.56)	
**Year**					0.009
	**2009**	363(6.25)	336(6.03)	27(11.54)	
	**2010**	358(6.17)	349(6.26)	9(3.85)	

	**2011**	408(7.03)	388(6.96)	20(8.55)	
	**2012**	466(8.03)	442(7.93)	24(10.26)	
	**2013**	471(8.11)	447(8.02)	24(10.26)	
	**2014**	518(8.92)	505(9.06)	13(5.56)	
	**2015**	538(9.27)	520(9.33)	18(7.69)	
	**2016**	631(10.87)	611(10.97)	20(8.55)	
	**2017**	653(11.25)	633(11.36)	20(8.55)	
	**2018**	653(11.25)	626(11.23)	27(11.54)	
	**2019**	747(12.87)	715(12.83)	32(13.68)	
**Parity**					0.3
	**1**	3126(53.84)	2996(53.77)	130(55.56)	
	**2**	2041(35.15)	1968(35.32)	73(31.2)	
	**3**	529(9.11)	505(9.06)	24(10.26)	
	**≥4**	102(1.76)	95(1.7)	7(2.99)	
	**Missing**	8(0.14)	8(0.14)	0	
**Premature birth**					<0.001
	**no**	3136(54.01)	3102(55.67)	34(14.53)	
	**yes**	2670(45.99)	2470(44.33)	200(85.47)	
**Hypertension**					0.9
	**no**	5616(96.73)	5390(96.73)	226(96.58)	
	**yes**	190(3.27)	182(3.27)	8(3.42)	
**Surgical indication**					<0.001
	**no**	1023(17.62)	931(16.71)	92(39.32)	
	**yes**	4783(82.38)	4641(83.29)	142(60.68)	
**Complication**					<0.001
	**no**	3641(62.71)	3545(63.62)	96(41.03)	
	**yes**	2165(37.29)	2027(36.38)	138(58.97)	
**Eclampsia**					0.4
	**no**	5452(93.9)	5235(93.95)	217(92.74)	
	**yes**	354(6.1)	337(6.05)	17(7.26)	
**GBS infection**					>0.9

**no**	5797(99.84)	5563(99.84)	234(100)	
**yes**	9(0.16)	9(0.16)	0	
**Anemia**				0.6
**no**	5721(98.54)	5489(98.51)	232(99.15)	
**yes**	85(1.46)	83(1.49)	2(0.85)	
**Antenatal care utilization rate**				<0.001
**≤50%**	989(17.03)	894(16.04)	95(40.6)	
**50-80%**	2206(38)	2112(37.9)	94(40.17)	
**80-110%**	969(16.69)	944(16.94)	25(10.68)	
**＞110%**	1642(28.28)	1622(29.11)	20(8.55)	
**Education**				0.005
**middle and high schools**	3856(66.41)	3697(66.35)	159(67.95)	
**Primary schools and below**	106(1.83)	95(1.7)	11(4.7)	
**University degree or above**	1844(31.76)	1780(31.95)	64(27.35)	
**Assisted reproductive techniques**				>0.9
**no**	5457(93.99)	5237(93.99)	220(94.02)	
**yes**	349(6.01)	335(6.01)	14(5.98)	
**High risk**				0.013
**no**	2229(38.39)	2121(38.07)	108(46.15)	
**yes**	3577(61.61)	3451(61.93)	126(53.85)	
**Husband smoking**				0.9
**no**	630(10.85)	610(10.95)	20(8.55)	
**yes**	506(8.72)	489(8.78)	17(7.26)	
**Missing**	4670(80.43)	4473(80.28)	197(84.19)	0.7
**Husband drinking**				
**no**	710(12.23)	689(12.37)	21(8.97)	
**yes**	386(6.65)	373(6.69)	13(5.56)	0.8
**Missing**	4710(81.12)	4510(80.94)	200(85.47)	
**Folic acid in the first trimester**				
**no**	4633(79.8)	4445(79.77)	188(80.34)	
**yes**	1173(20.2)	1127(20.23)	46(19.66)	0.8

**Abnormal pregnancy**					
	**no**	5112(88.05)	4907(88.07)	205(87.61)	
	**yes**	694(11.95)	665(11.93)	29(12.39)	
**Delivery way**					<0.001
	**cesarean section**	4846(83.47)	4731(84.91)	115(49.15)	
	**vaginal delivery**	953(16.41)	836(15)	117(50)	
	**Missing**	7(0.12)	5(0.09)	2(0.85)	
**Position**					0.7
	**LOA**	4380(75.44)	4202(75.41)	178(76.07)	
	**LSA**	469(8.08)	450(8.08)	19(8.12)	
	**ROA**	147(2.53)	141(2.53)	6(2.56)	
	**RSA**	89(1.53)	85(1.53)	4(1.71)	
	**Tire**	60(1.03)	57(1.02)	3(1.28)	
	**else**	371(6.39)	362(6.5)	9(3.85)	
	**Missing**	290(4.99)	275(4.94)	15(6.41)	
**Bad pregnancy and childbirth history**					0.8
	**no**	5112(88.05)	4907(88.07)	205(87.61)	
	**yes**	694(11.95)	665(11.93)	29(12.39)	
**History of spontaneous abortion**					0.8
	**no**	5197(89.51)	4989(89.54)	208(88.89)	
	**yes**	609(10.49)	583(10.46)	26(11.11)	
**History of neonatal death**					>0.9
	**no**	5777(99.5)	5544(99.5)	233(99.57)	
	**yes**	29(0.5)	28(0.5)	1(0.43)	
**History of birth defects**					>0.9
	**no**	5797(99.84)	5563(99.84)	234(100)	
	**yes**	9(0.16)	9(0.16)	0	

* Fisher's exact test ; Pearson's Chi-squared test ; R×C Chi-squared test

Table S4. Difference analysis for multiple births with birth at 5 min apgar score in Baoan Shenzhen, 2009-2019

	Total	non-asphyxiation	Asphyxiation	p*

	(N=5804,%)	(N=5728,%)	(N=76,%)	
**Maternal age**				0.12
**＜19**	30(0.52)	29(0.51)	1(1.32)	
**19~23**	208(3.58)	202(3.53)	6(7.89)	
**23~35**	4511(77.72)	4458(77.83)	53(69.74)	
**35~40**	839(14.46)	826(14.42)	13(17.11)	
**≥40**	216(3.72)	213(3.72)	3(3.95)	
**Year**				
**2009**	363(6.25)	351(6.13)	12(15.79)	
**2010**	358(6.17)	357(6.23)	1(1.32)	
**2011**	408(7.03)	400(6.98)	8(10.53)	
**2012**	466(8.03)	459(8.01)	7(9.21)	
**2013**	470(8.1)	462(8.07)	8(10.53)	
**2014**	517(8.91)	514(8.97)	3(3.95)	
**2015**	538(9.27)	533(9.31)	5(6.58)	
**2016**	631(10.87)	625(10.91)	6(7.89)	
**2017**	653(11.25)	647(11.3)	6(7.89)	
**2018**	653(11.25)	643(11.23)	10(13.16)	
**2019**	747(12.87)	737(12.87)	10(13.16)	
**Parity**				0.2
**1**	3125(53.84)	3078(53.74)	47(61.84)	
**2**	2040(35.15)	2021(35.28)	19(25)	
**3**	529(9.11)	520(9.08)	9(11.84)	
**≥4**	102(1.76)	101(1.76)	1(1.32)	
**Missing**	8(0.14)	8(0.14)	0	
**Premature birth**				<0.001
**no**	3136(54.03)	3125(54.56)	11(14.47)	
**yes**	2668(45.97)	2603(45.44)	65(85.53)	
**Hypertension**				0.071
**no**	5614(96.73)	5543(96.77)	71(93.42)	
**yes**	39(0.67)	37(0.65)	2(2.63)	
**yes**	151(2.6)	148(2.58)	3(3.95)	
**Surgical indication**				<0.001
**no**	1022(17.61)	991(17.3)	31(40.79)	

	**yes**	4782(82.39)	4737(82.7)	45(59.21)	
**Complication**					<0.001
	**no**	3641(62.73)	3609(63.01)	32(42.11)	
	**yes**	2163(37.27)	2119(36.99)	44(57.89)	
**Eclampsia**					0.8
	**no**	5450(93.9)	5379(93.91)	71(93.42)	
	**yes**	354(6.1)	349(6.09)	5(6.58)	
**Gbs infection**					>0.9
	**no**	5795(99.84)	5719(99.84)	76(100)	
	**yes**	9(0.16)	9(0.16)	0	
**Anemia**					0.3
	**no**	5719(98.54)	5645(98.55)	74(97.37)	
	**yes**	85(1.46)	83(1.45)	2(2.63)	
**Antenatal care utilization rate**					<0.001
	**⩽50%**	987(17.01)	955(16.67)	32(42.11)	
	**50-80%**	2206(38.01)	2173(37.94)	33(43.42)	
	**80-110%**	969(16.7)	963(16.81)	6(7.89)	
	**＞110%**	1642(28.29)	1637(28.58)	5(6.58)	
**Education**					0.025
	**middle and high schools**	3854(66.4)	3805(66.43)	49(64.47)	
	**primary schools and below**	106(1.83)	101(1.76)	5(6.58)	
	**university degree or above**	1844(31.77)	1822(31.81)	22(28.95)	
**Assisted reproductive techniques**					>0.9
	**no**	5455(93.99)	5383(93.98)	72(94.74)	
	**yes**	349(6.01)	345(6.02)	4(5.26)	
**High risk**					0.3
	**no**	2227(38.37)	2193(38.29)	34(44.74)	
	**yes**	3577(61.63)	3535(61.71)	42(55.26)	
**Husband smoking**					0.5
	**no**	630(10.85)	626(10.93)	4(5.26)	
	**yes**	506(8.72)	501(8.75)	5(6.58)	
	**Missing**	4668(80.43)	4601(80.32)	67(88.16)	
**Husband drinking**					0.5
	**no**	710(12.23)	704(12.29)	6(7.89)	

yes	386(6.65)	384(6.7)	2(2.63)	
Missing	4708(81.12)	4640(81.01)	68(89.47)	
**Folic acid in the first trimester**				0.7
no	4631(79.79)	4569(79.77)	62(81.58)	
yes	1173(20.21)	1159(20.23)	14(18.42)	
**Abnormal pregnancy**				0.08
no	5110(88.04)	5048(88.13)	62(81.58)	
yes	694(11.96)	680(11.87)	14(18.42)	
**Delivery way**				<0.001
cesarean section	4845(83.48)	4809(83.96)	36(47.37)	
vaginal delivery	952(16.4)	913(15.94)	39(51.32)	
Missing	7(0.12)	6(0.1)	1(1.32)	
**Position**				0.7
LOA	4378(75.43)	4324(75.49)	54(71.05)	
LSA	469(8.08)	465(8.12)	4(5.26)	
ROA	147(2.53)	146(2.55)	1(1.32)	
RSA	89(1.53)	87(1.52)	2(2.63)	
Tire	60(1.03)	60(1.05)	0	
else	371(6.39)	365(6.37)	6(7.89)	
Missing	290(5)	281(4.91)	9(11.84)	
**Bad pregnancy and childbirth history**				0.08
no	5110(88.04)	5048(88.13)	62(81.58)	
yes	694(11.96)	680(11.87)	14(18.42)	
**History of spontaneous abortion**				0.058
no	5195(89.51)	5132(89.59)	63(82.89)	
yes	609(10.49)	596(10.41)	13(17.11)	
**History of neonatal death**				>0.9
no	5775(99.5)	5699(99.49)	76(100)	
yes	29(0.5)	29(0.51)	0	
**History of birth defects**				>0.9
no	5795(99.84)	5719(99.84)	76(100)	
yes	9(0.16)	9(0.16)	0	

* Fisher's exact test ; Pearson's Chi-squared test ; R×C Chi-squared test

Table S5. Difference analysis with VBLW of multiple births in Baoan Shenzhen, 2009-2019

		Total (N=5818,%)	non-VBLW (N=5474,%)	VBLW (N=344,%)	p*
**Maternal age**					0.01
	**<19**	30(0.52)	26(0.47)	4(1.16)	
	**19~23**	211(3.63)	190(3.47)	21(6.1)	
	**23~35**	4519(77.67)	4247(77.58)	272(79.07)	
	**35~40**	842(14.47)	803(14.67)	39(11.34)	
	**≥40**	216(3.71)	208(3.8)	8(2.33)	
**Year**					0.057
	**2009**	363(6.24)	337(6.16)	26(7.56)	
	**2010**	361(6.2)	342(6.25)	19(5.52)	
	**2011**	411(7.06)	377(6.89)	34(9.88)	
	**2012**	467(8.03)	441(8.06)	26(7.56)	
	**2013**	476(8.18)	445(8.13)	31(9.01)	
	**2014**	518(8.9)	493(9.01)	25(7.27)	
	**2015**	538(9.25)	512(9.35)	26(7.56)	
	**2016**	631(10.85)	599(10.94)	32(9.3)	
	**2017**	653(11.22)	626(11.44)	27(7.85)	
	**2018**	653(11.22)	614(11.22)	39(11.34)	
	**2019**	747(12.84)	688(12.57)	59(17.15)	
**Parity**					0.04
	**1**	3132(53.83)	2926(53.45)	206(59.88)	
	**2**	2043(35.12)	1934(35.33)	109(31.69)	
	**3**	532(9.14)	512(9.35)	20(5.81)	
	**≥4**	103(1.77)	96(1.75)	7(2.03)	
	**Missing**	8(0.14)	6(0.11)	2(0.58)	
**Premature birth**					<0.001
	**no**	3138(53.94)	3132(57.22)	6(1.74)	
	**yes**	2680(46.06)	2342(42.78)	338(98.26)	
**Hypertension**					0.5
	**no**	5627(96.72)	5296(96.75)	331(96.22)	
	**yes**	39(0.67)	38(0.69)	1(0.29)	

	**yes**	152(2.61)	140(2.56)	12(3.49)	
**Surgical indication**					<0.001
	**no**	1033(17.76)	900(16.44)	133(38.66)	
	**yes**	4785(82.24)	4574(83.56)	211(61.34)	
**Complication**					<0.001
	**no**	3645(62.65)	3517(64.25)	128(37.21)	
	**yes**	2173(37.35)	1957(35.75)	216(62.79)	
**Eclampsia**					0.061
	**no**	5464(93.92)	5149(94.06)	315(91.57)	
	**yes**	354(6.08)	325(5.94)	29(8.43)	
**GBS infection**					0.1
	**no**	5809(99.85)	5467(99.87)	342(99.42)	
	**yes**	9(0.15)	7(0.13)	2(0.58)	
**Anemia**					0.3
	**no**	5733(98.54)	5392(98.5)	341(99.13)	
	**yes**	85(1.46)	82(1.5)	3(0.87)	
**Antenatal care utilization rate**					<0.001
	**⩽50%**	994(17.08)	857(15.66)	137(39.83)	
	**50-80%**	2212(38.02)	2062(37.67)	150(43.6)	
	**80-110%**	970(16.67)	943(17.23)	27(7.85)	
	**＞110%**	1642(28.22)	1612(29.45)	30(8.72)	
**Education**					0.075
	**middle and high schools**	3867(66.47)	3627(66.26)	240(69.77)	
	**primary schools and below**	106(1.82)	96(1.75)	10(2.91)	
	**university degree or above**	1845(31.71)	1751(31.99)	94(27.33)	
**Assisted reproductive techniques**					0.3
	**no**	5469(94)	5141(93.92)	328(95.35)	
	**yes**	349(6)	333(6.08)	16(4.65)	
**High risk**					<0.001
	**no**	2237(38.45)	2056(37.56)	181(52.62)	
	**yes**	3581(61.55)	3418(62.44)	163(47.38)	
**Husband smoking**					0.8
	**no**	631(10.85)	604(11.03)	27(7.85)	
	**yes**	506(8.7)	486(8.88)	20(5.81)	
	**Missing**	4681(80.46)	4384(80.09)	297(86.34)	
**Husband drinking**					0.4

**no**	711(12.22)	685(12.51)	26(7.56)	
**yes**	386(6.63)	368(6.72)	18(5.23)	
**Missing**	4721(81.14)	4421(80.76)	300(87.21)	
**Folic acid in the first trimester**				0.032
**no**	4643(79.8)	4353(79.52)	290(84.3)	
**yes**	1175(20.2)	1121(20.48)	54(15.7)	
**Abnormal pregnancy**				0.9
**no**	5124(88.07)	4822(88.09)	302(87.79)	
**yes**	694(11.93)	652(11.91)	42(12.21)	
**Delivery way**				<0.001
**cesarean section**	4846(83.29)	4672(85.35)	174(50.58)	
**vaginal delivery**	965(16.59)	796(14.54)	169(49.13)	
**Missing**	7(0.12)	6(0.11)	1(0.29)	
**Position**				0.097*
**else**	371(6.38)	357(6.52)	14(4.07)	
**LOA**	4384(75.35)	4118(75.23)	266(77.33)	
**LSA**	469(8.06)	439(8.02)	30(8.72)	
**ROA**	147(2.53)	142(2.59)	5(1.45)	
**RSA**	89(1.53)	83(1.52)	6(1.74)	
**Tire**	60(1.03)	60(1.1)	0	
**Missing**	298(5.12)	275(5.02)	23(6.69)	
**Bad pregnancy and childbirth history**				0.9
**no**	5124(88.07)	4822(88.09)	302(87.79)	
**yes**	694(11.93)	652(11.91)	42(12.21)	
**History of spontaneous abortion**				0.9
**no**	5209(89.53)	4900(89.51)	309(89.83)	
**yes**	609(10.47)	574(10.49)	35(10.17)	
**History of neonatal death**				0.7
**no**	5789(99.5)	5447(99.51)	342(99.42)	
**yes**	29(0.5)	27(0.49)	2(0.58)	
**History of birth defects**				>0.9
**no**	5809(99.85)	5465(99.84)	344(100)	
**yes**	9(0.15)	9(0.16)	0	

* Fisher's exact test ; Pearson's Chi-squared test ; R×C Chi-squared test

Table S6. Difference analysis analysis for multiple births with Height / head circumference ratio in Baoan Shenzhen, 2009-2019

	Total	normal	abnormal	P*
	(N=5787,%)	(N=5410,%)	(N=377,%)	
**Maternal age**				0.3
**＜19**	29(0.5)	27(0.05)	2(0.53)	
**19~23**	208(3.59)	187(0.35)	21(5.57)	
**23~35**	4499(77.74)	4208(7.78)	291(77.19)	
**35~40**	836(14.45)	786(1.45)	50(13.26)	
**≥40**	215(3.72)	202(0.37)	13(3.45)	
**Year**				0.2
**2009**	351(6.07)	320(0.59)	31(8.22)	
**2010**	355(6.13)	324(0.6)	31(8.22)	
**2011**	405(7)	374(0.69)	31(8.22)	
**2012**	467(8.07)	431(0.8)	36(9.55)	
**2013**	474(8.19)	442(0.82)	32(8.49)	
**2014**	517(8.93)	479(0.89)	38(10.08)	
**2015**	537(9.28)	507(0.94)	30(7.96)	
**2016**	631(10.9)	592(1.09)	39(10.34)	
**2017**	651(11.25)	615(1.14)	36(9.55)	
**2018**	653(11.28)	618(1.14)	35(9.28)	
**2019**	746(12.89)	708(1.31)	38(10.08)	
**Parity**				0.2
**1**	3113(53.79)	2894(5.35)	219(58.09)	
**2**	2036(35.18)	1920(3.55)	116(30.77)	
**3**	529(9.14)	492(0.91)	37(9.81)	
**≥4**	101(1.75)	96(0.18)	5(1.33)	
**Missing**	8(0.14)	8(0.01)	0(0)	
**Premature birth**				<0.001
**no**	3128(54.05)	3008(5.56)	120(31.83)	
**yes**	2659(45.95)	2402(4.44)	257(68.17)	
**Hypertension**				0.2

	**no**	5596(96.7)	5227(9.66)	369(97.88)	
	**yes**	191(3.3)	183(0.34)	8(2.12)	
**Surgical indication**					0.006
	**no**	1016(17.56)	930(1.72)	86(22.81)	
	**yes**	4771(82.44)	4480(8.28)	291(77.19)	
**Complication**					<0.001
	**no**	3625(62.64)	3435(6.35)	190(50.4)	
	**yes**	2162(37.36)	1975(3.65)	187(49.6)	
**Eclampsia**					0.5
	**no**	5434(93.9)	5083(9.39)	351(93.1)	
	**yes**	353(6.1)	327(0.6)	26(6.9)	
**GBS infection**					0.11
	**no**	5778(99.84)	5403(9.99)	375(99.47)	
	**yes**	9(0.16)	7(0.01)	2(0.53)	
**Anemia**					0.8
	**no**	5702(98.53)	5331(9.85)	371(98.41)	
	**yes**	85(1.47)	79(0.15)	6(1.59)	
**Antenatal care utilization rate**					<0.001
	**≤50%**	971(16.78)	862(1.59)	109(28.91)	
	**50-80%**	2208(38.15)	2060(3.81)	148(39.26)	
	**80-110%**	966(16.69)	919(1.7)	47(12.47)	
	**>110%**	1642(28.37)	1569(2.9)	73(19.36)	
**Education**					0.6
	**middle and high schools**	3843(66.41)	3591(6.64)	252(66.84)	
	**primary schools and below**	104(1.8)	95(0.18)	9(2.39)	
	**university degree or above**	1840(31.8)	1724(3.19)	116(30.77)	
**Assisted reproductive techniques**					0.2
	**no**	5438(93.97)	5089(9.41)	349(92.57)	
	**yes**	349(6.03)	321(0.59)	28(7.43)	
**High risk**					0.3
	**no**	2217(38.31)	2063(3.81)	154(40.85)	

yes	3570(61.69)	3347(6.19)	223(59.15)	
**Husband smoking**				0.5
no	630(10.89)	587(1.08)	43(11.41)	
yes	507(8.76)	477(0.88)	30(7.96)	
Missing	4650(80.35)	4346(8.03)	304(80.64)	
**Husband drinking**				0.7
no	711(12.29)	663(1.23)	48(12.73)	
yes	386(6.67)	362(0.67)	24(6.37)	
Missing	4690(81.04)	4385(8.1)	305(80.9)	
**Folic acid in the first trimester**				0.3
no	4613(79.71)	4320(7.98)	293(77.72)	
yes	1174(20.29)	1090(2.01)	84(22.28)	
**Abnormal pregnancy**				0.8
no	5098(88.09)	4764(8.81)	334(88.59)	
yes	689(11.91)	646(1.19)	43(11.41)	
**Delivery way**				<0.001
cesarean section	4829(83.45)	4548(8.41)	281(74.54)	
vaginal delivery	951(16.43)	855(1.58)	96(25.46)	
Missing	7(0.12)	7(0.01)	0(0)	
**Position**				0.6
LOA	4369(75.5)	4100(7.58)	269(71.35)	
LSA	466(8.05)	430(0.79)	36(9.55)	
ROA	147(2.54)	136(0.25)	11(2.92)	
RSA	88(1.52)	81(0.15)	7(1.86)	
Tire	60(1.04)	56(0.1)	4(1.06)	
else	369(6.38)	349(0.65)	20(5.31)	
Missing	288(4.98)	258(0.48)	30(7.96)	
**Bad pregnancy and childbirth history**				0.8
no	5098(88.09)	4764(8.81)	334(88.59)	
yes	689(11.91)	646(1.19)	43(11.41)	
**History of spontaneous abortion**				0.7
no	5183(89.56)	4843(8.95)	340(90.19)	

	**yes**	604(10.44)	567(1.05)	37(9.81)	
**History of neonatal death**					0.7
	**no**	5758(99.5)	5383(9.95)	375(99.47)	
	**yes**	29(0.5)	27(0.05)	2(0.53)	
**History of birth defects**					0.5
	**no**	5778(99.84)	5402(9.98)	376(99.73)	
	**yes**	9(0.16)	8(0.01)	1(0.27)	

* Fisher's exact test ; Pearson's Chi-squared test ; R×C Chi-squared test

2. Supplementary Figures

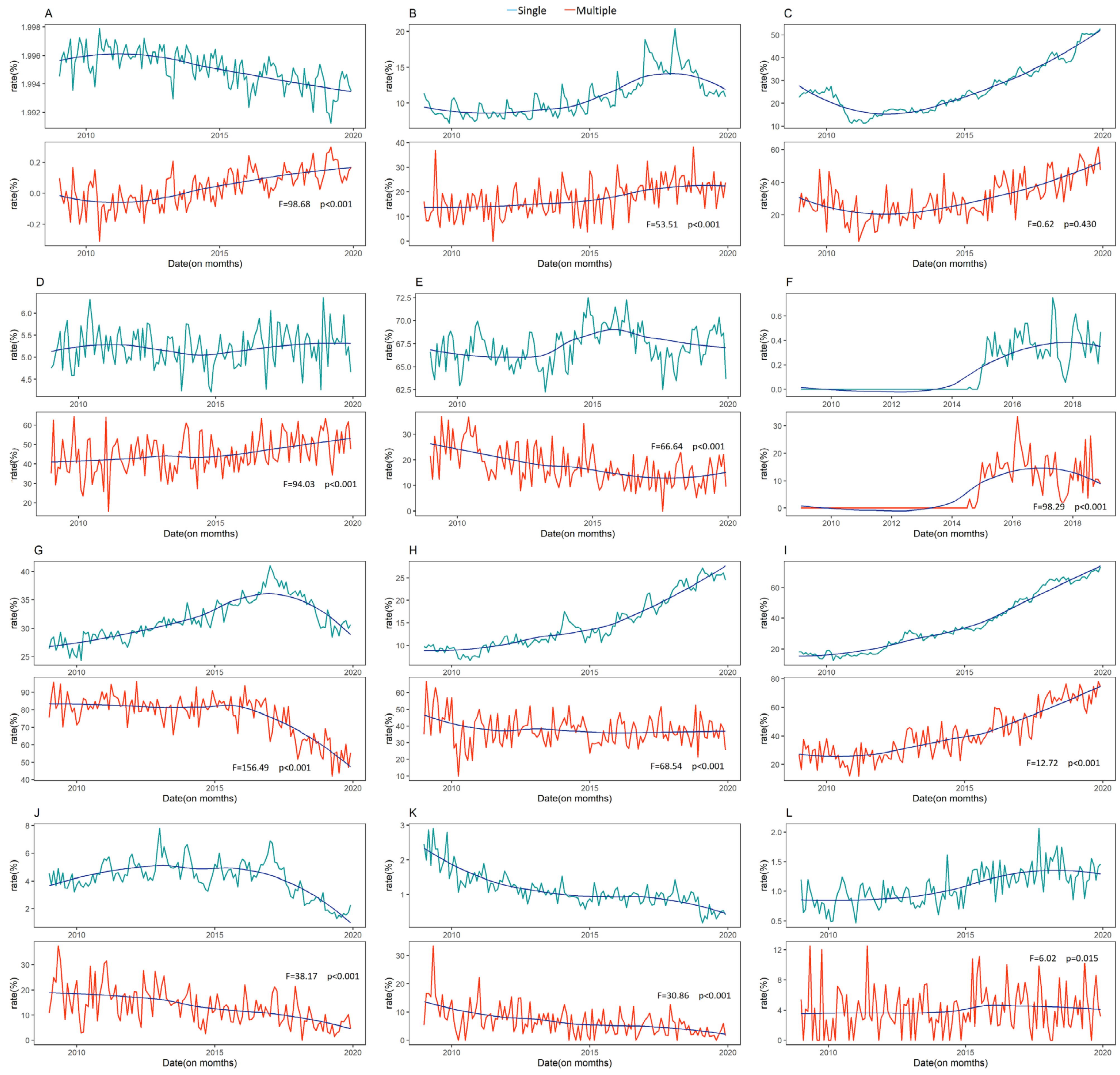

Figure S1. Sliding-window time series models of maternal socioeconomic and biomedical characteristics among 520,860 participants sub-categorized by singletons and multiple births in Baoan, Shenzhen, 2009-2019. (A) Natality, (B) Maternal age (proportion over 35 years old), (C) Education level, (D) Premature birth rate (<37week), (E) Vaginal delivery rate, (F) Pregnancy rate by assisted reproductive technology, (G) High risk factors during pregnancy, (H) Maternal complications rate, (I) Prenatal care utilization rate >80% during pregnancy, (J) Antenatal hypertension (K) Eclampsia rate, (L) Postpartum hemorrhage rate.

F & p: the differences in trend curve slopes by compare the interaction term in the generalized linear model. F: F-value, p: p-value.

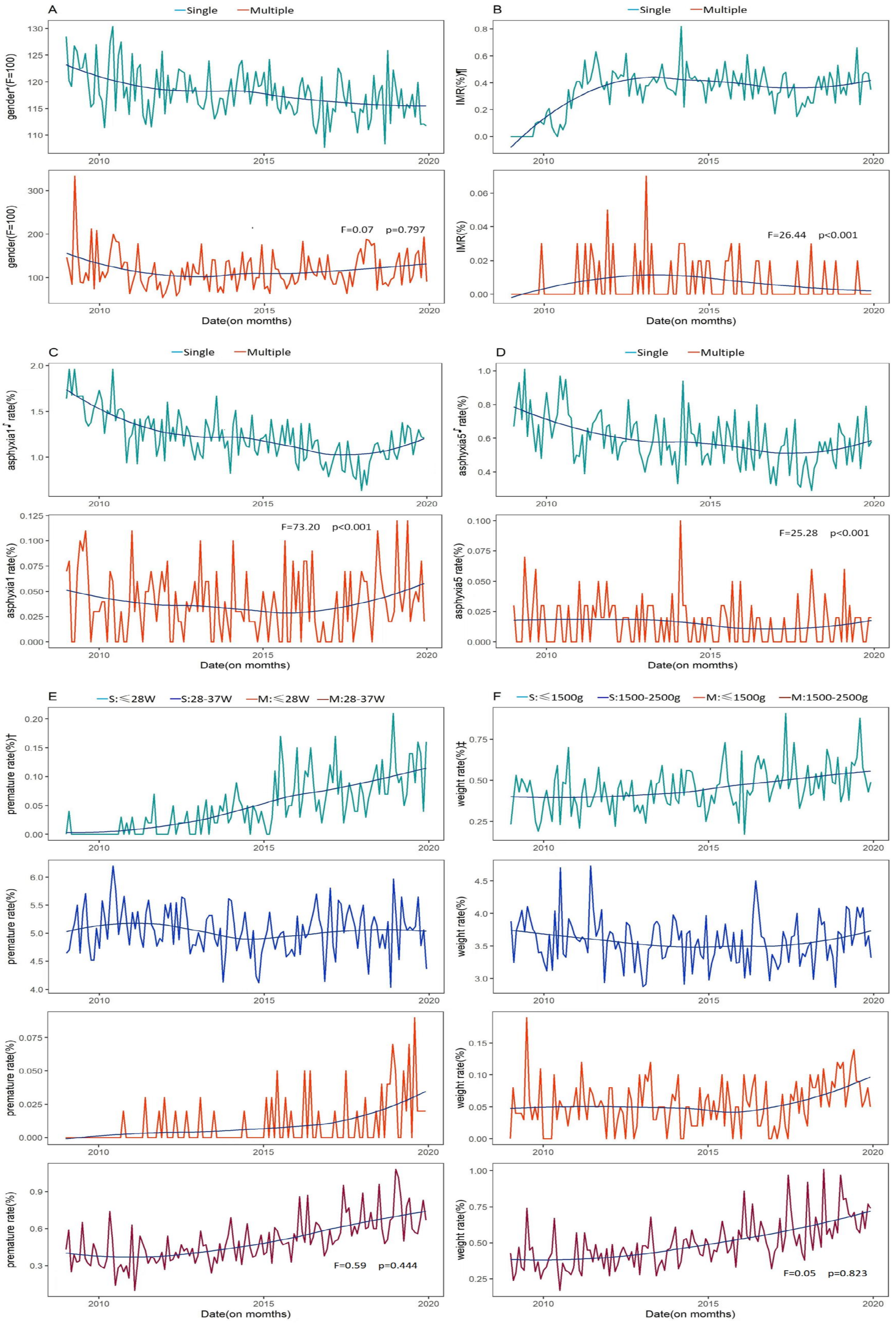

A
Single
Multiple
gender(F=100)
F=0.07 p=0.797
Date(on momths)
B
IMR(%)
F=26.44 p<0.001
C
asphyxia1 rate(%)
F=73.20 p<0.001
D
asphyxia5 rate(%)
F=25.28 p<0.001
E
S:≤28W
S:28-37W
M:≤28W
M:28-37W
premature rate(%)
F=0.59 p=0.444
F
S:≤1500g
S:1500-2500g
M:≤1500g
M:1500-2500g
weight rate(%)
F=0.05 p=0.823

Figure S2. Sliding-window time series models of the demographic and developmental characteristics among 526,654 newborns in Baoan, Shenzhen, 2009-2019. (A) gender ratio, (B) Infant Mortality Rate (IMR), (C) Asphyxia rate with apgar score <7 at one minute after birth, (D) Asphyxia rate with apgar score <7 at five minutes after birth, (E)Neonatal gestational age, (F) Neonatal weight.

F & p: the differences in trend curve slopes by compare the interaction term in the generalized linear model. F: F-value, p: p-value.

*: 526,654 newborns includes 515,016 singletons and 11,638 multiple births

¶:Cumulative mortality rate in 0-7 days

♪:Hypoxia assessed by Apgar scale in the first and fifth minutes after delivery of the fetus

†: Infant mortality refers to the death within 0-7 days of birth.

‡: M: multiple births, S: single birth
